# Effectiveness of antenatal screening of asymptomatic bacteriuria in reduction of prematurity and low birth weight: Evaluating a point-of-care rapid test in a pragmatic randomized controlled study

**DOI:** 10.1016/j.eclinm.2021.100762

**Published:** 2021-03-02

**Authors:** Manish Gehani, Suman Kapur, Sudha D Madhuri, Vara Prasad Pittala, Sree Kala Korvi, Nagamani Kammili, Shashwat Sharad

**Affiliations:** a Department of Biological Sciences, Birla Institute of Technology and Science, Pilani, Hyderabad Campus, Hyderabad 500078, India; b Department of Microbiology, Gandhi Medical College and Hospital, Secunderabad 500003, India; c Jhpiego, Hyderabad 500038, India; d Independent Consultant, Hyderabad, 500038, India; e Center for Prostate Disease Research, John P. Murtha Cancer Center Research Program, Department of Surgery, Uniformed Services University of the Health Sciences and Walter Reed National Military Medical Center, Bethesda, MD, United States

**Keywords:** Rapid test, Asymptomatic bacteriuria, Low birth weight, Pragmatic randomized controlled trial, Pregnant women, Preterm birth, Point-of-care test

## Abstract

**Background:**

Premature babies suffer higher mortality and life-long disabilities. Asymptomatic bacteriuria (ASB) is postulated to induce preterm labor. Routine antenatal screening for ASB using urine culture is not feasible in most developing countries due to long turn-around time, user-unfriendliness, and lack of resources. The current parallel-group superiority pragmatic randomized controlled trial evaluated the effect of screening and evidence-based treatment of ASB using an optical-sensor-based point-of-care rapid-test on the incidence of preterm birth and low birthweight (LBW).

**Methods:**

240 consenting asymptomatic pregnant women visiting an Indian tertiary public hospital for first antenatal check-up, irrespective of trimester/gravida, who had not consumed antibiotics in the preceding week, were enrolled from February-May 2017. Computer-generated concealed simple randomization allocation sequence was used to assign participants to intervention (120) and control arm (120). Usual hospital-care was provided in the control arm. In the intervention arm, urine samples were additionally screened for ASB using the rapid-test and the positive women were prescribed susceptible antibiotics. Blinded outcome assessors followed up with women post-delivery. The study was registered with the Clinical Trials Registry-India (CTRI/2016/09/007240).

**Findings:**

213 participants were analyzed (intervention: 103, control: 110). 21 women were found positive for ASB and prescribed pathogen-specific antibiotics. The incidence of preterm birth/LBW in intervention arm (*n* = 27) was lower than control arm (*n* = 45) by 14·7% (95% CI: 2·2–27·2); RR: 0.64, (95% CI: 0·43–0·95); *p* = 0·023, X^2^=5·13.

**Interpretation:**

Rapid-test-guided treatment for ASB reduced the incidence of preterm birth/LBW in a pragmatic setting without any adverse event.

**Funding:**

Department of Biotechnology, Government of India.

**Research in context**

**Evidence before this study**

Conventional urine culture was not shown to be useful in mass screening of pregnant women for asymptomatic bacteriuria (ASB). The alternative tests tried in the past to screen ASB could not provide identification and antibiotic susceptibility test (ID & AST) and did not possess characteristics suitable for screening mass population, namely, rapidity, validity, user-friendliness, and affordability. No study had measured the effectiveness of a point-of-care test on reduction of preterm birth and low birthweight (LBW) in a real-life situation.

**Added value of this study**

The study showed the effectiveness of use of a rapid point-of-care test during antenatal checkup in reducing preterm birth and LBW by informing clinicians regarding ID & AST for ASB within four hours. The study also proved feasibility of using a rapid, user-friendly, affordable, and valid test for mass screening of ASB in pregnant women.

**Implications of all the available evidence**

The rapid point-of-care test evaluated in this study can be used for mass screening of ASB in pregnant women in low resource settings so as to provide evidence-based prescription to pregnant women instead of empirical antibiotics. This can also reduce antimicrobial resistance. The confirmation of test results can be done using conventional urine culture, but the patient need not wait for up to 72 h for initiation of effective therapy using antibiotics.

Alt-text: Unlabelled box

## 1 Introduction

Pregnant women are susceptible to colonization of the urinary tract like asymptomatic bacteriuria (ASB), or urogenital infections, such as bacterial vaginosis, vulvovaginal candidiasis, and urinary tract infections (UTI), which co-exist in many cases [1,2]. As a result of the biochemical mechanisms operating in these conditions [3,4], the women may experience preterm labor. Preterm birth and its consequent complications contribute 50% of 3·1 million neonatal deaths world-wide annually, while it is also noteworthy that India contributes 23% of the world's annual premature deliveries [5]. Of all the deaths caused by low birth weight (LBW)/prematurity, more than 70% occur during the first week of life, highlighting the importance of continuum of care starting from the early antenatal period to the early neonatal period [6]. Even if the babies survive after preterm birth, its long-term adverse effects related to visual, hearing, neuro-development, behavior, psychiatric issues, learning disability, lung maturity and cardiovascular health continue in most of them throughout life, also burdening their families and health system due to the need of the long-term special care [5].

The progress towards achieving the Sustainable Development Goals (SDG) target 3·2 of reducing preventable deaths of newborns and children under 5 years of age [7] has been slower due to multiple factors including lack of cause-specific solutions [8]. Since preterm birth contributes to majority of these deaths, and the proportion of deaths due to LBW/prematurity has remained high in spite of intensive efforts done during antenatal care [6], focusing on the preventable causes of preterm birth such as ASB is a promising solution. Out of ASB-affected pregnancies, which account for nearly 1·9 to 9·5% of 213 million estimated pregnancies in a year worldwide [9], around 8·6% to 13·6% end in preterm birth, while 6·9% to 20·5% end in LBW [10,11]. Early diagnosis and treatment of ASB can reduce preterm birth among these pregnancies by 73%, and LBW by 36% [12], which extrapolate to potentially reduce 0·25 to 2 million preterm births and 0·1 to 1·5 million LBW babies globally and 57,375 to 453,660 preterm births and 22,701 to 337,229 LBW in India every year.

Antenatal check-ups provide an opportunity to detect ASB. Infectious Diseases Society of America (IDSA) [9] and United States Preventive Service Task Force (USPSTF) [13] recommend urine culture for screening every pregnant woman for ASB at least once between 12 and 16-weeks’ gestation or during the first antenatal visit. Most pregnant women in low- and middle-income countries (LMIC) like India attend antenatal check-ups in low resource healthcare facilities in rural areas, which suffer from wide disparity in availability and accessibility to health infrastructure and diagnostic testing facilities, and fail to provide screening for ASB as per the recommendations [14,15]. Conventional urine culture is not suitable for this purpose considering the need for specific resources, dedicated infrastructure, trained personnel, and long turnaround time [16]. USPSTF suggested to develop a new technology for rapid screening to reduce the dependence on conventional urine culture and improve the outcomes of such pregnancies [13]. Similar use of rapid point-of-care tests in primary care has been found to be beneficial for many infectious diseases due to availability of bedside results in a short turnaround time in remote areas with limited infrastructure [17]. Several tests have been tried to screen ASB but none of them provided identification of bacteria and antimicrobial sensitivity (ID & AST) or possessed characteristics like rapidity, validity, user-friendliness, and affordability [16]. The rapid test employed in this study is based on optical sensors and provides results of ID & AST within four hours at the point-of-testing [18]. This novel test has been clinically validated in around 2000 patients suffering from UTI [18].

The process of development of prematurity and LBW due to ASB is largely insidious and influenced by an interplay of multiple factors [5]. Variability is also introduced at facility-level, including training status of lab technicians, resource availability, adherence to guidelines, myths among the providers regarding asymptomatic nature of the disease, and lack of habit of conducting point-of-care tests in routine antenatal check-ups. Hence, usefulness of an intervention targeted to reduce the burden of prematurity and LBW attributable to early detection and informed management of ASB cannot be studied in close confine of laboratories and needs a pragmatic real-life set up with minimal control, which is provided by a pragmatic randomized controlled trial design. Such a design has been widely used for generating evidence of effectiveness of interventions in actual clinical setting [19].

The objective of the current study was to evaluate the effect of early detection and evidence-based treatment of ASB in pregnancy using this rapid point-of-care test on incidence of preterm birth and LBW in a real-world situation.

## 2 Methods

### 2.1 Ethical approval

The study was reviewed and approved by Institutional Ethics Committee of Gandhi Medical College, Secunderabad, India. Participants were enrolled after explaining the details of the study in their native language and obtaining written informed consent. The study was conducted according to the principles expressed in the Declaration of Helsinki.

### 2.2 Study design and setting

**Fig. 1:**
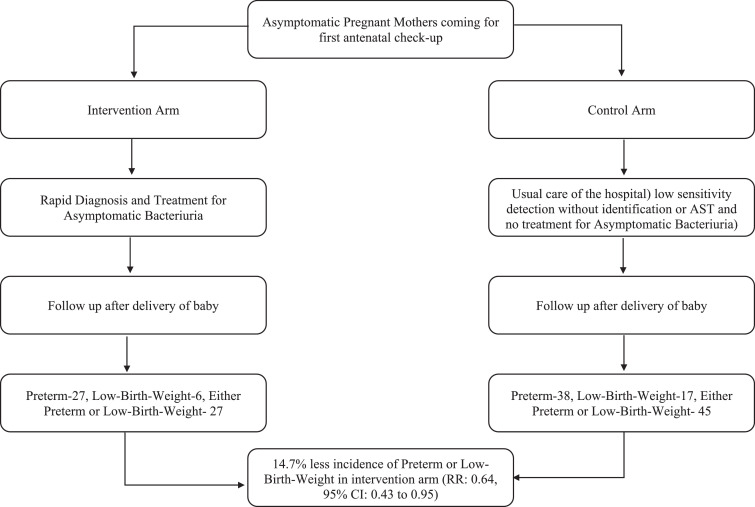
Care pathways evaluated in the study. Sequence of events and outcomes in the two arms- intervention and the control arm.

The study was conducted in a parallel-group pragmatic randomized controlled design (Fig. 1) with 1:1 allocation, to identify the superiority of the intervention- “using a rapid test and prescribing antibiotics based on positive antimicrobial sensitivity results”, over the usual care in the study hospital in case of ASB. Randomization would have minimized the effect of other factors affecting preterm birth and LBW. Unit of randomization was pregnant woman.

To help achieve the required sample size and to facilitate the resource mobilization for the study site, the study was conducted in a government tertiary care hospital, Gandhi Hospital, Secunderabad, India, from February 2017 to February 2018. The study hospital receives walk-in patients mostly from the areas within and around Secunderabad and Hyderabad, which are twin cities in the state of Telangana, India with a combined population of nearly 700,000, and a reported prevalence of ASB in pregnancy as 18% [21]. Referred patients from even other districts of Telangana are also catered by the hospital since it is a tertiary care hospital. Thus, the participants in both arms are representative of a wider population. As the participants were selected from a pool or line-list of clients who visited the study hospital based on their own preference and choice, the results may not be generalized to the entire population of the city or state.

The reader machine for the novel test was installed in the microbiology laboratory of Gandhi Hospital by Department of Biological Sciences, Birla Institute of Technology and Science (BITS) Pilani, Hyderabad Campus, for the complete duration of the study. Strips for the test were supplied by the Genomics Laboratory at BITS Pilani. The research staff involved were lab technicians from BITS Pilani and Obstetricians and Microbiologists from Gandhi Hospital.

The study was registered with the Clinical Trials Registry-India (ID number CTRI/2016/09/007240). The study protocol is provided as an appendix (Appendix 1).

### 2.3 Study population, sampling, eligibility and enrolment

The study population consisted of the pregnant women attending antenatal clinic of the hospital for first antenatal check-up irrespective of trimester and gravida. Every day nearly 50 to 150 women used to visit the antenatal clinic, who were first registered in an antenatal register. Randomx android mobile app was used to select the participants by simple random sampling of the line list of the register for that day. The consenting participants were evaluated clinically by a research team consisting of a doctor and a lab technician, and those with any sign or symptom of UTI were excluded. Any participant who had a history of consumption of antibiotics in the preceding week was also excluded. Based on the mission capacity of the study team, maximum ten participants per day and on an average four participants per day were enrolled on weekdays until the sample size was met. Identification details, antenatal history, inclusion/exclusion criteria, and clinical evaluation details were recorded in a pre-approved data collection format. A urine sample was collected in a sterile container from each of the participants.

### 2.4 Randomization and allocation concealment

The urine samples were sequentially numbered before sending them to the microbiology laboratory. Computerized random allocation sequence was generated by Dr Manish Gehani using Microsoft Excel's random number generation feature, which was concealed from the team members involved in enrolling and assessing participants in the antenatal clinic. Following simple randomization method without any restriction, participants were randomly assigned in microbiology laboratory to either intervention (*n* = 120) or control (*n* = 120) group. Selectively, the urine samples from only the intervention arm were provided to the novel test team for further processing.

### 2.5 Intervention and usual care in control arm

Participants were not informed about their allocation to either intervention or control arm. All the participants, irrespective of the arm in which they were enrolled, underwent routine first antenatal check-up in antenatal out-patient department (OPD) [22], which included history taking, general and obstetric examination, and routine blood and urine tests. The battery of tests conducted during routine antenatal check-up did not include urine culture and sensitivity or rapid test but included routine urine examination and microscopy [22]. Even for pus cells more than ten per high power field, the obstetricians did not order urine culture and sensitivity, and did not prescribe any antibiotics, since the patient was asymptomatic. In control arm, this usual care of hospital for asymptomatic pregnant women coming for their first antenatal check-up was continued without any interference.

Whereas, pregnant women enrolled in intervention arm were additionally screened for ASB with the novel rapid test [18,23,24]. The rapid test utilized a proprietary medium to promote growth of uropathogens; chromogens to impart color to the medium; a pre-functionalized identification strip; two pre-loaded antibiotic strips; and a reader machine with optical sensor to detect chromogenic and nephelometric endpoints, and a proprietary indigenous lab-developed statistical algorithm-based software. For each test, 10 ml of urine sample was harvested in 3 ml of the proprietary medium at room temperature for 5 min and then the resultant suspension was added to the strips and incubated. Subsequently, the strips were kept in the reader machine for generating software-based results. Performing each test took a turn-around-time of four hours. The machine provided at the same time, the results of bacterial load; direct quantitative detection of common UTI-causing bacteria found in human urine, namely *Escherichia coli, Klebsiella, Pseudomonas, Enterococcus, Proteus, and Staphylococcus* sp.; and phenotypic antibiotic sensitivity of the causative bacteria to Amoxicillin, Gentamicin, Amikacin, Cefepime, Ofloxacin, Ciprofloxacin, Ceftriaxone, Piperacillin-Tazobactum, Cefotaxime, Cefuroxime, Tobramycin, Levofloxacin, Cefazolin and Imipenem [18,23,24]. The sensitivity and specificity of the rapid test was found to be 92.5% and 82% respectively as compared to the gold standard urine culture [18].

The positive results of ID & AST were shared immediately with obstetricians on duty to assist them in clinical decision making and in prescribing the standard regimen and duration of course of a freely chosen antibiotic based on the AST report of the rapid test, during the same hospital visit of the participant. Telephonic follow-up was done for only those women in the intervention arm who were prescribed antibiotics after being found positive for ASB. They were called on day 1, 3 and 7 after their hospital visit and prescription of antibiotics, to ensure compliance to the prescribed antibiotic regimen.

### 2.6 Follow-up and masking of data collectors

The length of follow up was till the delivery of baby. All 240 women enrolled in both the arms were followed up after delivery of baby, by three graduate data collectors (outcome assessors) experienced in field epidemiology, who were blinded regarding the allocation of the participants. They called each participant on her Expected Date of Delivery (EDD) and visited her in the hospital where she gave birth to the child. Based on the interview of the patient and the information recorded in the case record and discharge record by hospital nurse and verified by obstetrician, the data collectors recorded in a pre-designed format, the date of delivery, gestational age at childbirth and birth weight. In case the participant could not be contacted or traced around the EDD, or had delivered before the initial contact around EDD, they were visited at their home and data was collected by interview and by reviewing the discharge record. History was also taken of other complications of pregnancy and of symptomatic UTI after first antenatal check-up.

### 2.7 Outcome measures

The primary outcome measure was a delivered baby having either preterm birth or LBW or both. As per WHO definition, preterm birth is defined as babies born alive before 37 completed weeks of pregnancy [5], while LBW is defined as weight at birth being less than 2500 gs [25]. The secondary outcome of the study was the incidence of other maternal and perinatal morbidities, irrespective of whether hospital admissions were needed for them, or not needed for mild complaints which could be managed without admission. The chosen outcome is important to the experts and decision makers who recommend and implement guidelines, as preterm birth and LBW affect child survival and additional resources are needed to cater special care to such vulnerable infants.

### 2.8 Materials

Analytical-grade chemicals required for preparation of BITGEN (proprietary growth promoting media), and identification strips were procured from Sigma Chemicals, USA. The 8-well strips and syringe filters were procured from NUNC, Denmark, and sterile syringes from Dispovan, India. The scanner/reader machine for novel test was obtained from Micro Lab Instruments, India.

### 2.9 Statistical analysis

For sample size calculation, the global rate of preterm birth, 10·6%, and that of LBW, 14·6%, were combined to extrapolate the combined rate of preterm or LBW (primary outcome measure) as 25%, which was assumed to be the incidence in the control arm. Based on the contribution of ASB to total preterm birth and LBW, which varies from 6·9% to 20·5% [20], the expected incidence of preterm birth or LBW in intervention arm, was safely assumed to be 15% lower than the control arm, as a minimally important reduction for success of the study. Based on type 1 error of 5%, power of 80%, and loss to follow up as 10% (5% in each arm), it was estimated that 240 participants should be enrolled in the study (120 in each arm). Pearson's chi square was used for analyzing data using SPSS (v24), after tabulation of categorical dichotomous outcome of preterm birth or LBW and the two arms in a 2 × 2 table. The cut off of p-value used in the study to indicate statistical significance was 0.05. Intention-to-treat analysis was planned in the original groups in which the participants were allocated. Incidence of other maternal and perinatal events was described as frequencies and proportions in two arms were compared for significance using z test.

### 2.10 Role of funding source

The funding agency had no role in design of the study, collection of data, collection of samples, processing of the rapid point-of-care test, analysis and interpretation of data, writing of paper or submission for publication. The study and researchers were independent of the funding agency. The authors had full control of all primary data at all times and accept responsibility to submit for publication.

## 3 Results

### 3.1 Participants

**Fig. 2:**
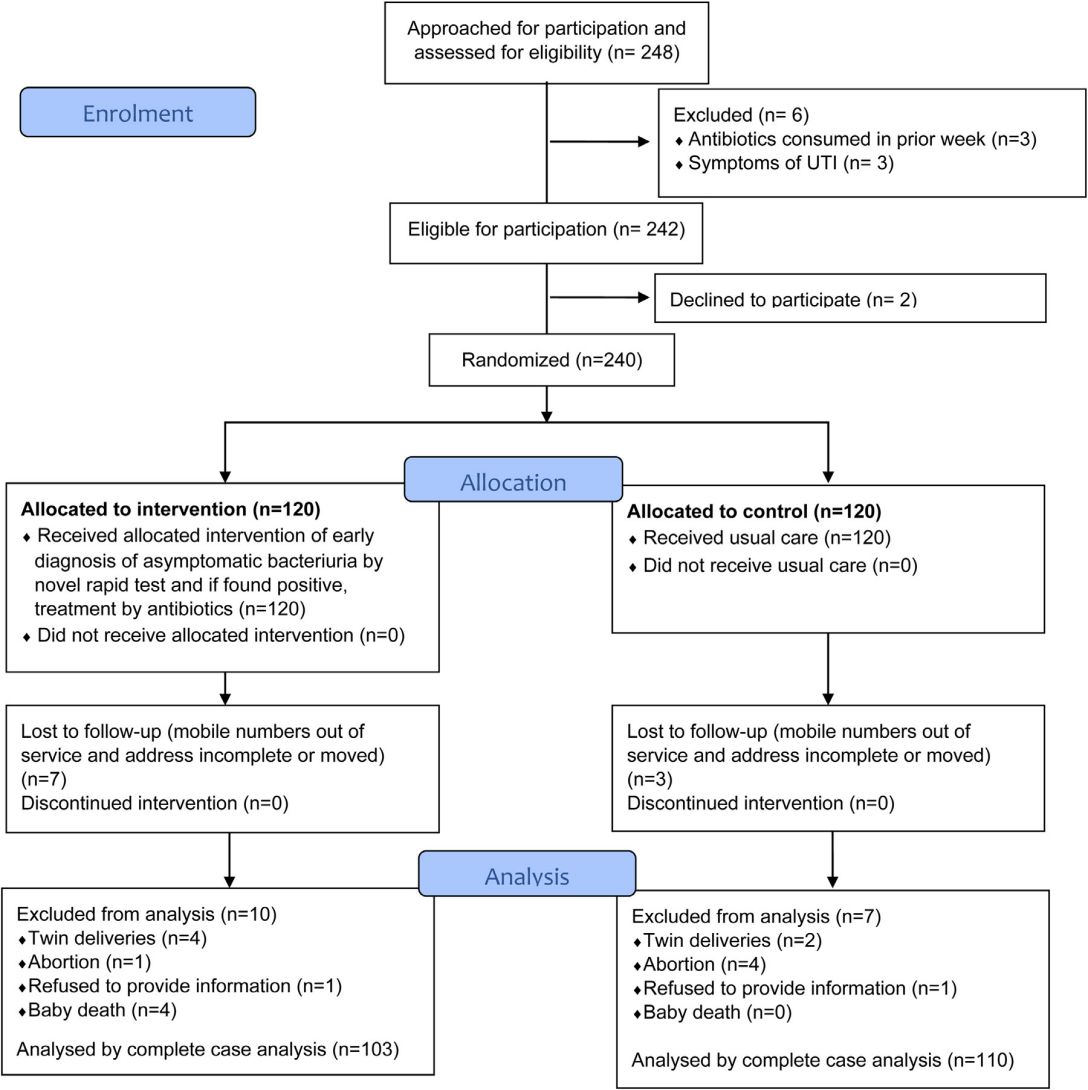
CONSORT diagram of flow of participants through the study (Trial Profile). A flow diagram of the study participants through the stages of enrolment, allocation, follow-up, and analysis.

Eligible participants were recruited from 15th February to 18th May 2017, while the follow-ups of women were done from February 2017 to February 2018, as and when they delivered. Since many women came for first check-up in their third trimester, the length of follow up period varied from one day to 287 days from the date of enrolment. 248 pregnant women were approached for participation in the study. 242 were eligible and 240 women agreed to participate. None of the women discontinued the intervention (Fig. 2).

### 3.2 Baseline characteristics of participants

Based on the baseline characteristics, the participants in both the arms were similar and any difference was a result of mere chance (Table 1). Since the proportion of participants in second or third trimester (79·1% in control arm versus 73·3% in intervention arm) and as multigravida (56·7% in control arm versus 50·8% in intervention arm), was only insignificantly higher in control arm, the prognostic strength and chance imbalance of these variables were low. Hence baseline risk of ASB due to these factors was comparable in two groups.

**Table 1:** Baseline characteristics of participants. Baselines characteristics of the enrolled participants with respect to socio-demographic and antenatal factors.

	Intervention (*n* = 120)	Control (*n* = 120)	Statistical Test and value	p value
Age				
Mean- Years (SD)^1^	23.2 (3.1)	23.8 (3.4)	Levene's test for equality of variance: p value = 0.426 Independent samples T test: Equal variance assumed	0.128
Range	18–36 years	17–35 years		
**Religion**			Pearson's Chi-square value = 3.179	0.204
Christian	6 (5.0%)	11 (9.2%)		
Hindu	88 (73.3%)	76 (63.3%)		
Muslim	26 (21.7%)	33 (27.5%)		
**Education**			Fisher-Freeman-Halton Exact Test value = 10.260	0.877
Illiterate	13 (10.8%)	9 (7.5%)		
Below Primary Level	2 (1.7%)	1 (0.8%)		
Primary	3 (2.5%)	4 (3.3%)		
6th to 9th	20 (16.7%)	24 (20%)		
Secondary	44 (36.7%)	43 (35.8%)		
Intermediate & Collegian	15 (12.5%)	18 (15.0%)		
Diploma	0	1 (0.8%)		
Graduate	22 (18.3%)	18 (15%)		
Post-graduate	1 (0.8%)	2 (1.7%)		
**Pregnancy**	120 (100%)	120 (100%)	NA	NA
**Asymptomatic**	120 (100%)	120 (100%)	NA	NA
**Trimester at the time of enrolment**			Pearson's Chi-square value = 1.525	0.466
First	32 (26.7%)	25 (20.8%)		
Second	73 (60.8%)	82 (68.3%)		
Third	15 (12.5%)	13 (10.8%)		
**Gravida**			Pearson's Chi-square value = 0.821	0.365
Primigravida	59 (49.2%)	52 (43.3%)		
Multigravida	61 (50.8%)	68 (56.7%)		

1 SD: Standard Deviation.

### 3.3 Test results and prescription of antibiotics

In total, 20 women in intervention arm had ASB, out of which *E. coli* was identified in nine women, *Enterococcus* in four, *Klebsiella* in three, *Pseudomonas* in one, and *Staphylococcus* in three. For gram negative bacteriuria, gentamycin was prescribed in five cases, cephalosporins in seven cases, and piperacillin-tazobactum in one case, while for gram positive bacteriuria, amoxycillin was prescribed in two cases and cephalosporins in five case. One woman in the intervention arm, who was negative at the time of enrolment, presented with signs and symptoms of UTI in her 29th week of gestation. On re-evaluation with the novel test, she was found to have bacteriuria due to *E. coli*, for which she was prescribed piperacillin-tazobactum. In this way, total 21 women received antibiotics in the intervention arm (20 for ASB and one for UTI). No participant in control arm received any antibiotic for ASB at the time of enrolment. Although, in due course of pregnancy, three women in control arm had suffered with symptomatic UTI and had received antibiotics for the same.

### 3.4 Outcome analysis

#### 3.4.1 Analysis of the primary outcome of the incidence of either preterm birth or lbw

When a participant was called on her EDD, there were cases when the telephone numbers were out of service or not reachable and no alternative numbers were available. After three trials of calling the participants on three consecutive days, if they were still not contactable, the address provided by them at the time of enrolment was visited. In case they were not traceable even after home visit, due to migration and lack of information with their neighbors, they were labeled as lost to follow up. Due to loss to follow-up (*n* = 10), abortions (*n* = 5), death of new-born (*n* = 4), twin deliveries (*n* = 6), and refusal to divulge any information (*n* = 2), the final analysis included 103 participants in intervention arm and 110 participants in control arm for complete case analysis. There was no deviation from protocol during the study. Out of 213 women, 212 had institutional deliveries, while one woman (in control arm) delivered at home. Among institutional deliveries, eight had caesarean section (two in intervention arm and six in control arm), while 204 had normal delivery. A 14·7% (95% CI: 2·2 to 27·2) lower incidence of preterm birth or LBW was observed in the intervention arm (27 cases in intervention arm and 45 in control arm) (RR: 0.64, 95% CI: 0·43 to 0·95) (Table 2). Six cases in intervention arm and ten cases in control arm had both preterm birth and LBW, while majority of preterm births were late preterm births. The gestational age at delivery for preterm births and LBW babies are provided in Supplementary Tables 1 and 2. Out of 21 women who received antibiotics in intervention arm, only four experienced preterm birth or LBW, while three were lost to follow up. A significant association was found between intervention and occurrence of preterm birth or LBW by pre-specified Pearson's chi square test (X^2^= 5·13, *p* = 0·023). No adverse events were reported due to the intervention. Early diagnosis enabled obstetricians with timely confirmation of bacteriuria and rapid antibiotic susceptibility helped them in prescribing antibiotics based on readily available evidence during the same visit.

**Table 2:** Primary outcomes of the study. Outcomes of preterm or low birth weight in both the arms of the study and their risk ratio and risk difference with chi square values and p values.

	Intervention arm (*n* = 103)	Control arm (*n* = 110)	Risk ratio (95% CI)	Risk difference% (95% CI)	Chi-square (p value)
Preterm Birth	27 (26.2%)	38 (34.5%)	0.76 (0.50 to 1.15)	−8.3 (−20.6 to −4.0)	1.741 (0.187)
Low Birth Weight	6 (5.8%)	17 (15.5%)	0.38 (0.16 to 0.92)	−9.7 (−17.8 to −1.5)	5.121 (0.024)
Preterm Birth or Low Birth Weight^1^	27 (26.2%)	45 (40.9%)	0.64 (0.43 to 0.95)	−14.7 (−27.2 to −2.2)	5.134 (0.023)

1 6 cases in intervention arm and 10 cases in control arm had both preterm birth and low-birth-weight babies.

#### 3.4.2 Secondary outcomes

Other complications of pregnancy were distributed in both the arms as shown in Table 3. Maternal and other perinatal complications reported were 15·53% and 7·8% in intervention arm respectively, and 15·45% and 2·7% in control arm respectively. No significant difference was found between intervention arm and control arm with respect to maternal (*p* = 0.9871) and perinatal (*p* = 0.0929) complications.

**Table 3:** Secondary outcomes of the study. Other morbidities reported at the time of follow-up.

	Frequency inintervention arm (*n* = 103)	Frequency incontrol arm (*n* = 110)	Statistical test and value	p value
**Maternal**	**16 (15.53%)**	**17 (15.45%)**	z test* for comparing two proportions: value = 0	0.9871
*Haemorrhage*				
Antepartum Haemorrhage	0	1		
Postpartum Haemorrhage	1	0		
*Hypertensive disorders of pregnancy*				
Unspecified hypertension	0	2		
Gestational hypertension	0	2		
Mild preeclampsia	1	0		
Severe preeclampsia	0	1		
Eclampsia	2	0		
*Others*				
Amniotic fluid aspiration	0	1		
Oligohydramnios	5	1		
Polyhydramnios	0	1		
Pelvic Inflammatory Disease	1	0		
Premature rupture of membrane	3	1		
Gestational diabetes	1	2		
Hypothyroidism	1	2		
Urinary tract infections	1	3		
**Other perinatal complications**	**8 (7.8%)**	**3 (2.7%)**	Z test* for comparing two proportions: value = 1.7	0.0929
Meconium aspiration	1	0		
Atrial septal defect	1	0		
Congenital heart disease	1	1		
Cord around neck	1	0		
Foetal distress with birth asphyxia	1	0		
Neonatal Jaundice	2	1		
Genu varum	0	1		
Oedema	1	0		

⁎ 2 tailed test since frequency of maternal or perinatal complications can be more in any group.

## 4 Discussion

To the best of our knowledge, this study is one of the first studies evaluating usability of a rapid point-of-care technology for ID and AST in real practice conditions for reducing preventable perinatal and neonatal mortality, lifelong disability and health expenditure due to preterm birth. The clinical implication of arresting disease processes by early detection using rapid diagnostics and subsequent timely and appropriate treatment is a widely recognized approach [26].

The confidence interval ranging from 2·2% to 27·2% less cases of preterm birth or LBW in intervention arm, indicates a clinically important effect of early diagnosis of ASB and its subsequent treatment with appropriate antibiotics. Since the value of chi-square (5·13) exceeds the critical value (3·84) for one degree of freedom, and p value is less than 0·05, we conclude that preterm birth and LBW were significantly higher in control arm than in intervention arm. Considering the inter-current events between randomization and point of analysis, randomization would have minimized the risk of confounding due to other causative factors, although this risk cannot necessarily be considered to be eliminated. Thus, it can safely be assumed that the observed increase in cases of preterm birth and LBW in the control arm were attributable to undetected and hence, untreated ASB.

Recently, Lee et al. conducted a cluster randomized trial to evaluate the effect of population-based antenatal screening using home-based urine culture analysis and empirical treatment of genitourinary tract infections on birth outcomes [27]. The screening of abnormal vaginal flora and UTI in the study was based on samples collected and transported by community health worker from patient's home, which had higher risk of contamination. Whereas our study was a randomized controlled trial with facility-based intervention where the sample processing was done immediately after collection. Moreover, everybody who had an abnormal vaginal flora was treated empirically by Lee et al. with clindamycin and those suspected of UTIs were initially treated with cefixime and later with nitrofurantoin, after a high prevalence of cefixime resistance was detected. It is worth mentioning here that the antibiotics administered to subjects by Lee et al. were not based on actual testing for antibiotic sensitivity of the bacteria found in the urine sample or vaginal swab. In contrast to this, the present study prescribed specific antibiotics to patients diagnosed with bacteriuria based on antibiotic sensitivity data, and thus assisted in prescription of effective antibiotics and avoid antimicrobial resistance. Consequently, Lee et al. could not attain the reduction in the incidence of preterm birth [27]. In a similar study, Kazemier et al. could not show association of ASB and preterm birth due to use of empirical nitrofurantoin prescription instead of pathogen specific antibiotic based on AST report [28]. Use of a single antimicrobial can have differential results as opposed to using a pathogen specific antibiotic based on AST results. The current study showed that the rapid-test-guided treatment for ASB effectively lowered the incidence of preterm birth and LBW in the intervention arm by 14·7%.

Previously published meta-analyses have shown a strong association between untreated ASB and the incidence of preterm birth or LBW [11]. The promising results obtained from the present study emphasize the usefulness of a rapid test over conventional urine culture and of susceptibility-based prescription of antibiotics over empirical prescription for antibiotics. The usefulness of the results of a hospital-based pragmatic randomized controlled trial increases considering the fact that pregnant women come to hospitals for antenatal check-ups and the rapid test can become the part of the usual care in the hospital.

There were certain limitations of the study. The estimation of gestational age was based on fundal height or recall of the last menstrual period. To reduce inaccuracy in measurement and recording of endpoints, the labor room staff at Gandhi Hospital were re-trained for obstetric examination, weight measurement and estimation of gestational age before commencing the study and calibration of the digital weighing scales used in the hospital was ensured. Another limitation was that blinding those women in intervention arm who had a positive result was not possible, since they needed prescription of antibiotics. Similarly, the rapid test team in the microbiology laboratory could also not be blinded, as they selectively screened the urine samples of the participants enrolled in the intervention arm. Moreover, the study team at antenatal clinic was also made aware of the positive results of the participants in order to facilitate the prescription of antibiotics for them and to follow up with them for compliance to the prescribed antibiotic regimen. Nevertheless, data was collected prior to this disclosure, follow-ups were done to ensure compliance to the protocol, and outcome assessors were blinded to safeguard against bias. Many signs and symptoms of UTI also occur normally in pregnancy, still any such feature was out-rightly excluded to enroll only completely asymptomatic women. In India, many women visit hospital for first antenatal checkup directly in third trimester. Due to the pragmatic nature of the study and to reflect the actual state of care as it is in the facility, women were enrolled even when they came for the first antenatal checkup in third trimester. This arrangement created a possibility that if a woman would have come for first antenatal checkup in 36 weeks, she would never have reached the endpoint of preterm birth. Although, the highest gestational age of any woman remained 32 weeks at the time of enrolment in the study. The study also did not control for early preterm or late preterm births. Six cases in intervention arm and ten cases in control arm had both preterm birth and LBW, which implies that majority of preterm births in both arms (21 in intervention arm and 28 in control arm) were late preterm births. The study was conducted in a tertiary care hospital to achieve the required sample size. Tertiary care hospitals are adequately staffed, well located and more resourceful, however it is noteworthy that still they are unable to provide regular urine culture testing for the large population routinely visiting these places for antenatal care.

The novel rapid test described herein has unique features like portability and point-of-use testing, user-friendliness, low cost and no dependence on lab infrastructure. It can be performed by minimally-trained healthcare workers. These features make it feasible to install and run this test in primary and secondary care government run peripheral rural and remote hospitals, especially in LMICs. Availability of ID & AST results by this test in a short time interval can enable the clinicians to prescribe antibiotics on basis of real evidence for efficacy of a given antibiotic on a case-to-case basis, and to follow guidelines like screening pregnant women routinely for ASB. It fulfills 25 out of 26 Delphi-consensus criteria for point-of-care test for UTI detection except for just one, i.e., use of small sample volume [29]. The novel test also demonstrates high user acceptance by fulfilling six out of seven of the WHO's (World Health Organization) ASSURED (Affordable, Sensitive, Specific, User-friendly, Rapid and robust, Equipment-free and Deliverable to end-users) criteria for a low-cost point-of-care test in resource-limited settings, the only unfulfilled criteria being equipment free [30]. Due to these advantages, it can be scaled for mass screening of pregnant women during antenatal check-ups.

In conclusion, it can be emphasized that the real-world application of the portable rapid tests has immense potential in clinical care. This should be further evaluated using a multi-centric approach in a larger cohort and included in established clinical algorithms and public policies for rapid diagnosis during routine check-ups.

## Author contributions

SK and SS conceptualized the study. SK and MG prepared study design. SDM and MG searched the literature. VPP, SKK, SDM and MG collected data. MG ran data analysis. SK, NK and MG verified and interpreted the data. MG, SK and SS wrote the first draft of the manuscript. Final editing of the manuscript was done by SS, SK, MG and NK. All authors have reviewed and approved the manuscript.

## Declaration of Competing Interest

Authors have no competing interests to disclose.

## Appendix Supplementary materials

Image, application 1

Image, application 2

Image, application 3
