# Supplementary material for: Effectiveness of antenatal screening of asymptomatic bacteriuria in reduction of prematurity and low birth weight: Evaluating a point-of-care rapid test in a pragmatic randomized controlled study
Source: eClinicalMedicine. 2021 Mar 2;33:100762. doi: 10.1016/j.eclinm.2021.100762 (PMC8020147; doi:10.1016/j.eclinm.2021.100762)
Supplement: Supplementary file 1 [file mmc1.docx]

**Supplementary Table 1:** Gestational age at delivery (in weeks) for preterm births

| **S. No.** | **Intervention Arm** | **S. No.** | **Control Arm** |
| --- | --- | --- | --- |
| 1 | 27.71 | 1 | 24.14 |
| 2 | 30.29 | 2 | 30 |
| 3 | 31.29 | 3 | 30.29 |
| 4 | 31.57 | 4 | 30.43 |
| 5 | 32.29 | 5 | 31.57 |
| 6 | 32.71 | 6 | 32.71 |
| 7 | 32.86 | 7 | 32.71 |
| 8 | 32.86 | 8 | 32.86 |
| 9 | 33.29 | 9 | 32.86 |
| 10 | 33.71 | 10 | 33.14 |
| 11 | 33.86 | 11 | 33.14 |
| 12 | 34 | 12 | 33.14 |
| 13 | 34 | 13 | 33.71 |
| 14 | 34.29 | 14 | 34 |
| 15 | 34.57 | 15 | 34.14 |
| 16 | 34.57 | 16 | 34.14 |
| 17 | 34.86 | 17 | 34.57 |
| 18 | 35.29 | 18 | 34.57 |
| 19 | 35.43 | 19 | 34.57 |
| 20 | 35.57 | 20 | 34.86 |
| 21 | 35.71 | 21 | 34.86 |
| 22 | 36 | 22 | 34.86 |
| 23 | 36 | 23 | 35 |
| 24 | 36.14 | 24 | 35 |
| 25 | 36.29 | 25 | 35.14 |
| 26 | 36.57 | 26 | 35.14 |
| 27 | 36.86 | 27 | 35.29 |
|  |  | 28 | 35.71 |
|  |  | 29 | 36 |
|  |  | 30 | 36 |
|  |  | 31 | 36.29 |
|  |  | 32 | 36.29 |
|  |  | 33 | 36.29 |
|  |  | 34 | 36.43 |
|  |  | 35 | 36.57 |
|  |  | 36 | 36.57 |
|  |  | 37 | 36.71 |
|  |  | 38 | 36.86 |
