# Supplementary material for: Effectiveness of antenatal screening of asymptomatic bacteriuria in reduction of prematurity and low birth weight: Evaluating a point-of-care rapid test in a pragmatic randomized controlled study
Source: eClinicalMedicine. 2021 Mar 2;33:100762. doi: 10.1016/j.eclinm.2021.100762 (PMC8020147; doi:10.1016/j.eclinm.2021.100762)
Supplement: Supplementary file 2 [file mmc2.docx]

**Supplementary Table 2:** Gestational age at delivery (in weeks) for low birth weight babies

| **S. No.** | **Intervention Arm** | **S. No.** | **Control Arm** |
| --- | --- | --- | --- |
| 1 | 31.29 | 1 | 24.14 |
| 2 | 32.29 | 2 | 30.29 |
| 3 | 33.86 | 3 | 30.43 |
| 4 | 34.86 | 4 | 31.57 |
| 5 | 35.29 | 5 | 34 |
| 6 | 36.29 | 6 | 34.14 |
|  |  | 7 | 34.57 |
|  |  | 8 | 35 |
|  |  | 9 | 35.29 |
|  |  | 10 | 36.71 |
|  |  | 11 | 37.71 |
|  |  | 12 | 38.14 |
|  |  | 13 | 39.29 |
|  |  | 14 | 40 |
|  |  | 15 | 40 |
|  |  | 16 | 40.86 |
|  |  | 17 | 41 |
