# Supplementary material for: Effectiveness of antenatal screening of asymptomatic bacteriuria in reduction of prematurity and low birth weight: Evaluating a point-of-care rapid test in a pragmatic randomized controlled study
Source: eClinicalMedicine. 2021 Mar 2;33:100762. doi: 10.1016/j.eclinm.2021.100762 (PMC8020147; doi:10.1016/j.eclinm.2021.100762)
Supplement: Supplementary file 3 [file mmc3.docx]

**Clinical Intervention Study
Protocol**

**(NCCIH 2012 Template)**

**FULL PROTOCOL TITLE**

A clinical investigation evaluating rapid strip test for screening bacteriuria among pregnant women and its effect on incidence of low birth weight and prematurity in new-borns: A pragmatic randomized controlled trial

**Study Chairman or Principal Investigator:**

Dr Manish Gehani,

MBBS, MBA, PhD Scholar,

Department of Biological Sciences,

BITS Pilani, Hyderabad Campus,

Shameerpet, Hyderabad,

Telangana- 500078

Tel: +91 40 6630 3563,

Fax: +91 40 6630 3998

**Supported by:**GYTI: SRISTI,

Biotechnology Industry Research Assistance Council,

Department of Biotechnology,

Government of India,

New Delhi-110003

Tel: +91 11 2438 9600

**Study Intervention Provided by:**

Department of Biological Sciences,

BITS Pilani, Hyderabad Campus,

Shameerpet, Hyderabad,

Telangana- 500078

**Tool Revision History**

Version Number: 1.0

Version Date: 01.05.2015

Summary of Revisions Made: Original Version

**TABLE OF CONTENTS**

*Page*

[**Clinical Intervention Study Protocol 1**](#_Toc55745808)

[**(NCCIH 2012 Template) 1**](#_Toc55745809)

[**FULL PROTOCOL TITLE 2**](#_Toc55745810)

[Tool Revision History 3](#_Toc55745811)

[**TABLE OF CONTENTS 4**](#_Toc55745812)

[STUDY TEAM ROSTER 7](#_Toc55745813)

[PARTICIPATING STUDY SITES 7](#_Toc55745814)

[PRÉCIS 8](#_Toc55745815)

[**1. STUDY OBJECTIVES 9**](#_Toc55745816)

[1.1 Primary Objective 9](#_Toc55745817)

[1.2 Secondary Objectives 9](#_Toc55745818)

[**2. BACKGROUND AND RATIONALE 9**](#_Toc55745819)

[2.1 Background on Condition, Disease, or Other Primary Study Focus 9](#_Toc55745820)

[2.2 Study Rationale 12](#_Toc55745821)

[**3. STUDY DESIGN 12**](#_Toc55745822)

[**4. SELECTION AND ENROLLMENT OF PARTICIPANTS 13**](#_Toc55745823)

[4.1 Inclusion Criteria 13](#_Toc55745824)

[4.2 Exclusion Criteria 14](#_Toc55745825)

[4.3 Study Enrollment Procedures 14](#_Toc55745826)

[**5. STUDY INTERVENTIONS 15**](#_Toc55745827)

[5.1 Interventions, Administration, and Duration 15](#_Toc55745828)

[5.2 Handling of Study Interventions 16](#_Toc55745829)

[5.3 Concomitant Interventions 16](#_Toc55745830)

[5.4 Adherence Assessment 16](#_Toc55745831)

[**6. STUDY PROCEDURES 16**](#_Toc55745832)

[6.1 Schedule of Evaluations 17](#_Toc55745833)

[6.2 Description of Evaluations 18](#_Toc55745834)

[6.2.1 Screening Evaluation 18](#_Toc55745835)

[6.2.2 Enrollment, Baseline, and/or Randomization 18](#_Toc55745836)

[6.2.3 Blinding 19](#_Toc55745837)

[6.2.4 Followup Visits 19](#_Toc55745838)

[6.2.5 Completion/Final Evaluation 19](#_Toc55745839)

[**7. SAFETY ASSESSMENTS 19**](#_Toc55745840)

[7.1 Specification of Safety Parameters 20](#_Toc55745841)

[7.2 Methods and Timing for Assessing, Recording, and Analyzing Safety Parameters 20](#_Toc55745842)

[7.3 Adverse Events and Serious Adverse Events 20](#_Toc55745843)

[7.4 Reporting Procedures 20](#_Toc55745844)

[7.5 Followup for Adverse Events 20](#_Toc55745845)

[7.6 Safety Monitoring 20](#_Toc55745846)

[**8. INTERVENTION DISCONTINUATION 20**](#_Toc55745847)

[**9. STATISTICAL CONSIDERATIONS 21**](#_Toc55745848)

[9.1 General Design Issues 21](#_Toc55745849)

[9.2 Sample Size and Randomization 21](#_Toc55745850)

[Treatment Assignment Procedures 22](#_Toc55745851)

[9.3 Definition of Populations 22](#_Toc55745852)

[9.4 Interim Analyses and Stopping Rules 22](#_Toc55745853)

[9.5 Outcomes 22](#_Toc55745854)

[9.5.1 Primary Outcome 22](#_Toc55745855)

[9.5.2 Secondary Outcomes 22](#_Toc55745856)

[9.6 Data Analyses 22](#_Toc55745857)

[**10. DATA COLLECTION AND QUALITY ASSURANCE 23**](#_Toc55745858)

[10.1 Data Collection Forms 23](#_Toc55745859)

[10.2 Data Management 23](#_Toc55745860)

[10.3 Quality Assurance 23](#_Toc55745861)

[10.3.1 Training 23](#_Toc55745862)

[10.3.2 Quality Control Committee 23](#_Toc55745863)

[10.3.3 Metrics 23](#_Toc55745864)

[10.3.4 Protocol Deviations 23](#_Toc55745865)

[10.3.5 Monitoring 23](#_Toc55745866)

[**11. PARTICIPANT RIGHTS AND CONFIDENTIALITY 24**](#_Toc55745867)

[11.1 Institutional Review Board (IRB) Review 24](#_Toc55745868)

[11.2 Informed Consent Forms 24](#_Toc55745869)

[11.3 Participant Confidentiality 24](#_Toc55745870)

[11.4 Study Discontinuation 24](#_Toc55745871)

[**12. COMMITTEES 24**](#_Toc55745872)

[**13. PUBLICATION OF RESEARCH FINDINGS 24**](#_Toc55745873)

[**14. REFERENCES 24**](#_Toc55745874)

[**15. SUPPLEMENTS/APPENDICES 27**](#_Toc55745875)

**STUDY TEAM ROSTER**

Dr. Suman Kapur- Department of Biological Sciences, BITS Pilani, Hyderabad Campus, Shameerpet, Hyderabad, Telangana- 500078

Tel: +91 40 6630 3563, +91 90102 02863 Fax: +91 40 6630 3998

Email: [skapur@hyderabad.bits-pilani.ac.in](mailto:skapur@hyderabad.bits-pilani.ac.in)

Dr Manish Gehani- Department of Biological Sciences, BITS Pilani, Hyderabad Campus, Shameerpet, Hyderabad, Telangana- 500078

Tel: +91 75688 74444

Email: [dr.manishgehani@gmail.com](mailto:dr.manishgehani@gmail.com)

Dr Sudha D Madhuri- Department of Microbiology, Gandhi Medical College and Hospital, Secunderabad

Tel: +91 94401 05871

Email: [Sudha_devara@yahoo.com](mailto:Sudha_devara@yahoo.com)

Dr Vara Prasad Pittala- Department of Maternal and Neonatal Health, Jhpiego, Hyderabad

Tel: +91 70326 50053

Email: [varamedconiihfw@gmail.com](mailto:varamedconiihfw@gmail.com)

**PARTICIPATING STUDY SITES**

Dr Nagamani Kammili- Department of Microbiology, Gandhi Medical College and Hospital, Secunderabad

Tel: +91 99665 33327

Email: [nagamaniy2k03@rediffmail.com](mailto:nagamaniy2k03@rediffmail.com)

Dr. Suman Kapur- Department of Biological Sciences, BITS Pilani, Hyderabad Campus, Shameerpet, Hyderabad, Telangana- 500078

Tel: +91 40 6630 3563, +91 90102 02863 Fax: +91 40 6630 3998

Email: [skapur@hyderabad.bits-pilani.ac.in](mailto:skapur@hyderabad.bits-pilani.ac.in)

**PRÉCIS**

**Study Title**

A clinical investigation evaluating rapid strip test for screening bacteriuria among pregnant women and its effect on incidence of low birth weight and prematurity in new-borns: A pragmatic randomized controlled trial

**Objectives**

To evaluate the effect of early detection and treatment of asymptomatic bacteriuria in pregnancy using a rapid point-of-care test on incidence of preterm birth and low birth weight

**Design and Outcomes**

The study will be a parallel-group superiority pragmatic randomized controlled trial with 1:1 allocation, to identify the superiority of the intervention of using a rapid test for informing clinical decision for prescribing antibiotics based on positive AST results, over the usual care in the study hospital in case of asymptomatic bacteriuria in pregnancy. Blinding will be done for outcome assessors.

The primary outcome of the study is occurrence of preterm birth or low birth weight or both as the outcome of pregnancy. The secondary outcome will be any other maternal or perinatal complication in the study participants.

**Interventions and Duration**

Intervention will be a rapid test involving the use of optical sensors to measure specific optical signal generated by the enzymatic hydrolysis of specific cocktails by uropathogens in a media optimized for promotion of their growth. The identification of the bacteria is based on specific chromogenic endpoints produced because of specific metabolic activity of each bacterial type. Measurement of inhibition of bacterial growth is done in the presence of specific antibiotic. The sensor output is analyzed using an indigenous software based on a statistical algorithm, pre-installed on a reader machine, which provides both the identification of the pathogen and its sensitivity to a panel of antibiotics within four hours at the point-of-care. Based on AST results, the obstetricians will be informed about the susceptible antibiotics for prescription.

The comparator will be the usual care of hospital for asymptomatic pregnant women coming for their first antenatal check-up including history taking, general and obstetric examination, and routine blood and urine tests which includes routine urine examination and microscopy. As reported by Gandhi Hospital, urine culture is not being done during the routine antenatal visit and no antibiotics is being prescribed for asymptomatic bacteriuria.

Intervention period will be for one day during the participants first antenatal visit and follow up will be done after delivery of baby to measure the outcomes.

**Sample Size and Population**

Sample size proposed for the study is 120 pregnant women in each of the intervention and control arm.

**1. STUDY OBJECTIVES**

**1.1 Primary Objective**

The early detection of asymptomatic bacteriuria with the help of rapid test and subsequent treatment by obstetrician is hypothesized to result in reduction of preterm birth and low birth. Therefore, the primary objective of the study will be to evaluate the effect of early detection and treatment of asymptomatic bacteriuria in pregnancy using a rapid point-of-care test on incidence of preterm birth and low birth weight

**1.2 Secondary Objectives**

The secondary objective will be to measure other maternal and perinatal complications occurring in the enrolled pregnant women so as to look for confounders if any.

**2. BACKGROUND AND RATIONALE**

**2.1 Background on Condition, Disease, or Other Primary Study Focus**

Urinary tract infection includes asymptomatic subclinical infection and symptomatic disease. It may involve any part of urinary tract like urethra, bladder, ureter, kidneys.

**Asymptomatic bacteriuria** in women is the presence of more than 100000 colony forming units of same bacterial strain in each mL of two consecutive urine samples collected by voided midstream clean-catch or at least 100 colony forming units per mL of catheterized specimen, in the absence of any symptoms of urinary tract infection (UTI) [1]. It is mostly caused by Escherichia coli, but Klebsiella pneumoniae, coagulase-negative Staphylococci, Enterococcus species, group B streptococci, and Gardnerella vaginalis are also reported in few cases [1].

**Uncomplicated urinary tract infection** in nonpregnant outpatient women without anatomic abnormalities or instrumentation of the urinary tract when involves lower urinary tract, it is called cystitis, while if it involves upper urinary tract, it is called pyelonephritis. The signs and symptoms of cystitis are- urinary frequency, urgency, dysuria, nocturia, supra-pubic discomfort, low-back pain, haematuria. The signs and symptoms of pyelonephritis are- flank pain, nausea, vomiting, fever (>38ºC), costo-vertebral angle tenderness, chills. Complicated urinary tract infection encompasses all other types of UTI.

Asymptomatic bacteriuria in pregnancy if untreated, may progress to cystitis or pyelonephritis, and may lead to adverse outcomes like preterm labour, hypertensive disease and preeclamptic toxemia, anaemia or post-partum endometritis [2, 3]. Acute pyelonephritis can also lead to transient renal failure, Acute Respiratory Distress Syndrome, sepsis, shock and haematological abnormalities [4]. In baby, it can lead to urinary tract infections in baby, low birth weight, intrauterine growth retardation, and higher perinatal mortality rate [2, 3, 5].

**Increased susceptibility to asymptomatic bacteriuria and UTI in Pregnancy**

The human body has various mechanisms to prevent the colonization of the urinary tract, like the anatomical structure of the urinary tract, the physical and chemical properties of the urine and the non-specific innate immunity of the body. There are also the mechanisms for release of anti-microbial substances such as Tamm-Horsfall protein, mucopolysaccharides, immunoglobulins IgA and IgG, lactoferrin, lipocalin, neutrophils, cytokines and antimicrobial peptides. Still pregnant women are highly susceptible to UTI and asymptomatic bacteriuria. Pregnant women develop ureteral dilatation and dilatation of renal pelvices and caliceal system, which sustains until the delivery, known as hydroureter and hydronephrosis of pregnancy. Urinary stasis and uretero-vesical reflux increase as a result of increased bladder volume and decreased bladder and ureteral tone. The physiological increase in plasma volume during pregnancy decreases urine concentration. Moreover, pregnant women also develop glycosuria, aminoaciduria and decrease in immunity, all of which increase the incidence of bacteriuria. Additionally, there is decreased ability of the lower urinary tract to resist the bacteria due to an increase in urinary progestins and estrogens. The position of rectum and urethra come close to each other. Thus the anatomical and functional changes in the urinary tract in pregnancy predispose pregnant women to UTI and asymptomatic bacteriuria [6-8].

**Prevalence**

Prevalence of asymptomatic bacteriuria in pregnant women is reported to be 1.9 to 9.5% [1, 9], while that of any form of Urinary tract infections in pregnant women is 2 to 10% [10]. Thus 90 to 95% of UTI in pregnant women is asymptomatic.

The various risk factors for increased prevalence of UTI or asymptomatic bacteriuria being- low socio-economic status, advanced maternal age, multi-parity, increasing period of gestation, poor sanitation, lack of general hygiene and failure to attend antenatal clinic [11-13].

**Screening**

Considering the adverse effects of asymptomatic bacteriuria and symptomatic urinary tract infections in pregnant women and on the outcome of pregnancy, and considering the increased susceptibility of pregnant women to these infections, and since the prevalence is as high as 10% in pregnant women, it has been recommended by US Preventive Services Task Force and Infectious Diseases Society of America to screen every pregnant woman at least once in early pregnancy.

Screening for asymptomatic bacteriuria and its subsequent treatment has shown to reduce the risk of pyelonephritis [1, 2, 14, 15], preterm delivery and low birth weight [1, 16, 17]. Meta analyses of cohort studies and randomized controlled trials also support the conclusion that antimicrobial treatment of asymptomatic bacteriuria decreases the frequency of low–birth weight infants and preterm delivery. The clinical and epidemiologic evidence in a meta-analysis has indicated a strong association between untreated asymptomatic bacteriuria and LBW/preterm delivery [1, 16, 17]. It has also shown that antibiotic treatment is effective in reducing the occurrence of LBW [1, 16, 17].

**International recommendations for screening**

**US Preventive Services Task Force recommendation-**

The USPSTF recommends screening for asymptomatic bacteriuria with urine culture for pregnant women at 12 to 16 weeks’ gestation or at the first prenatal visit, if later [18].

**Infectious Diseases Society of America recommendation-**

Pregnant women should be screened for bacteriuria by urine culture at least once in early pregnancy, and they should be treated if the results are positive [1].

**Current practice lacks implementation of guidelines**

The conventional culture test takes 48 to 72 hours when done manually, and 18 to 24 hours when automated. It is costly and not easy to use as it needs autoclave, laminar air flow, dedicated space and trained personnel. This has led to reserving the urine culture test for only symptomatic patients by clinicians. Usually hospitals also do not screen routinely the pregnant women by urine culture. Many hospitals do screening by urine routine and microscopy during ante-natal check-ups. Those patients, who show more than 10 pus cells per high power field, are then sent for urine culture. The method of urine microscopy has shown low sensitivity [19] and thus many pregnant women are missed from the diagnosis of asymptomatic bacteriuria and suffer from its adverse consequences. Due to the delay in getting the results of urine culture, the pregnant women fail to come back to the hospital second or third day, when the culture and sensitivity reports come, due to many factors like lack of conveyance, cost of transportation, lack of compliance and discomfort of repeated visits. Therefore doctors generally prescribe empirical antibiotics to the women found symptomatic and then change the antibiotics as per the sensitivity results if the patient comes back after two to three days. The prescription of empirical antibiotics has raised the antimicrobial resistance for several broad-spectrum antibiotics. If the sensitivity report shows sensitivity to antibiotics which are not safe in pregnancy, it inadvertently leads to prescription of potentially harmful antibiotics in pregnancy, such as use of chloramphenicol, fluoro-quinolones etc. Irrational and unnecessary drug use can be expensive and harmful [20].

**Tests used so far for screening for asymptomatic bacteriuria in various studies**

Several tests have been used to screen pregnant women for asymptomatic bacteriuria but none has been able to replace the gold standard test of Urine Culture and sensitivity. The tests tried by various investigators so far are- Enhanced urinanalysis, chlorhexidine, dipstick or multistix with leucocyte esterase and nitrite, gram-stained smear, dipslide test culture, urine microscopy, uricult trio, interleukin-8, griess test, dornfest method, serum procalcitonin level and microstix-3 [21-34]. Most of the tests had low validity and were not having diagnostic accuracy.

According to USPTF, “Research is also needed to develop a screening test that could reduce the use of urine culture, which is labour-intensive and more costly than other urine tests”.

It is clear from the above literature review that no novel test has been successful to show validity compared to classical urine culture method. There is a need of not only a valid test, but also an easy-to-use and rapid test. Although some of the authors have studied the incidence of various outcomes of bacteriuria and have studied various tests separately, but no one has studied the effect of the test and subsequent treatment on the reduction in incidence of the adverse outcomes of pregnancy. Moreover, no study has compared the new test with existing protocols of a hospital, so as to influence and implement the policy level changes.

**2.2 Study Rationale**

Asymptomatic bacteriuria in pregnancy if untreated, may progress to cystitis or pyelonephritis, and may lead to adverse outcomes like preterm labour, hypertensive disease and preeclamptic toxemia, anaemia or post-partum endometritis [2, 3]. Acute pyelonephritis can also lead to transient renal failure, Acute Respiratory Distress Syndrome, sepsis, shock and haematological abnormalities [4]. In baby, it can lead to urinary tract infections in baby, low birth weight, intrauterine growth retardation, and higher perinatal mortality rate [2, 3, 5]. In this way asymptomatic bacteriuria leads to adverse outcomes of pregnancy. In order to prevent the adverse outcomes and complications, it has been recommended to screen and treat both asymptomatic and symptomatic bacteriuria.

Screening for asymptomatic bacteriuria and its subsequent treatment has shown to reduce the risk of pyelonephritis [1, 2, 14, 15], preterm delivery and low birth weight [1, 16, 17]. Meta analyses of cohort studies and randomized controlled trials also support the conclusion that antimicrobial treatment of asymptomatic bacteriuria decreases the frequency of low–birth weight infants and preterm delivery. The clinical and epidemiologic evidence in a meta-analysis has indicated a strong association between untreated asymptomatic bacteriuria and LBW/preterm delivery [1, 16, 17]. It has also shown that antibiotic treatment is effective in reducing the occurrence of LBW [1, 16, 17].

**3. STUDY DESIGN**

The study will be a parallel-group superiority pragmatic randomized controlled trial with 1:1 allocation, to identify the superiority of the intervention of using a rapid test for informing clinical decision for prescribing antibiotics based on positive AST results, over the usual care in the study hospital in case of asymptomatic bacteriuria in pregnancy. Blinding will be done for outcome assessors.

The only difference between the intervention and control arm will be the information provided by the rapid test regarding the identification of bacteria and susceptibility of antibiotics.

The primary outcome of the study is incidence of preterm birth or low birth weight or both as the outcome of pregnancy. The secondary outcome is incidence of any other maternal or perinatal complication in the study participants.

The study population will be pregnant women attending antenatal clinic of the hospital for first antenatal check-up. The study will have two arms- intervention arm and control arm. In each arm, 120 participants will be enrolled.

The setting of conduct of study will be antenatal outpatient clinic and microbiology laboratory of Gandhi Medical College and Hospital, Secunderabad. The reader machine for the novel test will be installed in the microbiology laboratory of Gandhi Hospital by Department of Biological Sciences, Birla Institute of Technology and Science (BITS) Pilani, Hyderabad Campus. The strips for conducting the test will be provided by Genomics Laboratory, Department of Biological Sciences, BITS Pilani, Hyderabad Campus.

Anticipated duration of study is one year, including a variable follow up period depending upon the Expected Date of Delivery of pregnant women.

Unit of randomization will be pregnant woman. A suitable method of randomization will be adopted out of coin toss, lottery, toss of dice, shuffling cards, MS Excel etc. The randomization sequence will be concealed from the staff enrolling and assessing. Simple randomization method will be adopted to randomly assign participants to either intervention or control group without any restriction. The Principal Investigator will discard urine samples of participants of control arm and will provide urine sample of participants of intervention arm to the staff in laboratory conducting rapid test, as per the randomization sequence. The positive samples will be informed to obstetrician on duty and will be prescribed antibiotics as per the report of the rapid test.

Blinding will be done for only the outcome assessors, who will visit the participants after delivery, to collect data related to the outcomes.

**Figure 1: Schematic Trial Design**


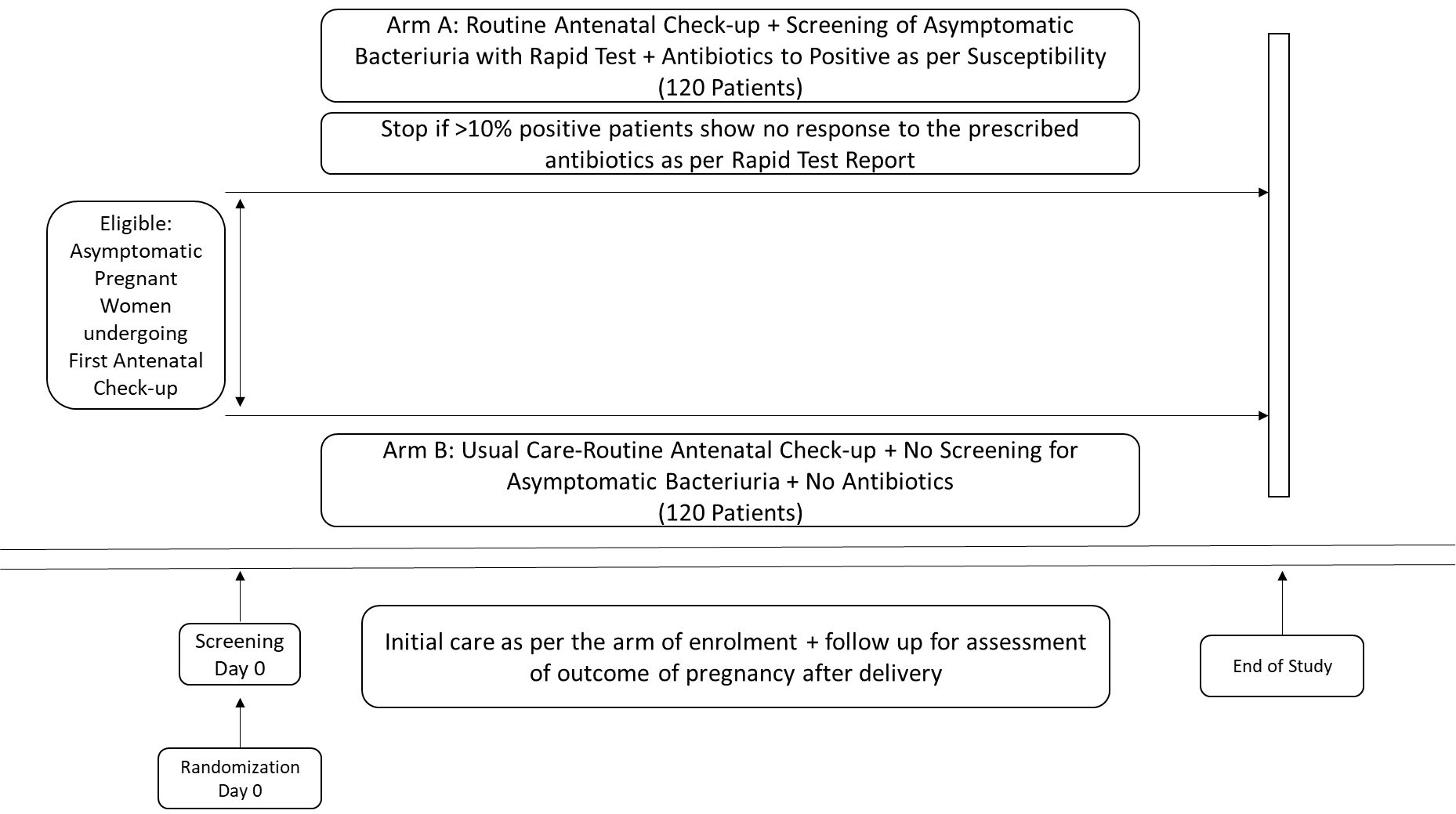


**4. SELECTION AND ENROLLMENT OF PARTICIPANTS**

Every day nearly 50 to 150 women visit the antenatal clinic for first antenatal checkup. These women are first registered in an antenatal register. Most of these women attending a Government Hospital have low socio-economic background. The study staff will use Randomx android mobile app to select the participants by simple random sampling of the line list of the register for that day. All the women attending antenatal clinic for their first antenatal checkup will have equal opportunity to get enrolled in the study due to simple random sampling. The women thus selected will be representative of the population of pregnant women accessing antenatal services in institutional setting. The results will have sufficient generalizability for such population in limited resource setting of low- middle- income countries. Based on the mission capacity of the novel test team, a suitable number participants will be enrolled every weekday until the sample size is met. Public holidays will be spared.

**4.1 Inclusion Criteria**

The participants must meet all of the inclusion criteria listed below to participate in this study-

1. Confirmed pregnancy based on urine pregnancy test or ultrasonography.

2. Pregnant woman irrespective of the age at the time of enrolment

3. Asymptomatic participant

4. Consenting and willing to provide a written informed consent.

5. Ability to understand study procedures and to comply with them for the entire length of the study

6. Any gravida or parity of woman

7. Pregnant woman coming in any trimester for first antenatal checkup as sometimes they come late during second or third trimester also.

8. History of antibiotic consumption more than one week ago

**4.2 Exclusion Criteria**

All candidates meeting any of the below-mentioned exclusion criteria at baseline will be excluded from the study participation:

1. History of consumption of antibiotics in the preceding week

2. Participants with any symptom/s out of urinary frequency, urgency, dysuria, nocturia, supra-pubic discomfort, low-back pain, haematuria, flank pain, nausea, vomiting, fever (99F), costo-vertebral angle tenderness or chills.

3. Inability or unwillingness of individual or legal guardian/representative to give written informed consent.

4. Participants enrolled in any other study

**4.3 Study Enrollment Procedures**

The study staff will use Randomx android mobile app to select the pregnant women coming to antenatal clinic for first antenatal checkup, by simple random sampling of the line list of the antenatal register for that day.

The selected participants will undergo informed consent procedure and if consented, a written informed consent will be signed in either English or vernacular language.

The participants will be screened for inclusion exclusion criteria based on history taking and physical assessment by the research staff for presence of symptoms. Identification details, antenatal history and clinical evaluation details will be recorded in a data collection tool. The data collection tool will also have the inclusion and exclusion criteria which will be ticked accordingly. A urine sample will be collected in sterile container from each of the participant and will be sequentially numbered before sending to the microbiology laboratory.

Based on the mission capacity of the novel test team, a suitable number participants will be enrolled every weekday until the sample size is met. Public holidays will be spared.

The urine samples will be sent to the microbiology laboratory where the Principal Investigator will selectively discard the samples from the control arm and will provide the samples from the intervention arm to the rapid test team as per the randomization allocation sequence.

All the enrolled participants irrespective of the arm in which they are enrolled, will be made to spend four hours from the time of urine sample collection. During this period, the participants of both intervention and control arm will undergo routine first antenatal check-up by obstetrician, which includes history taking, general and obstetric examination, and routine blood and urine tests (36). If they will finish the antenatal check-up early, they will be made to wait till the four hours are over. The waiting period is kept at four hours because the turn-around time of the rapid test is four hours. If the novel rapid test will come positive, the results will be shared with obstetricians on duty in the antenatal outpatient department for prescribing a susceptible obstetrician to the participant. The standard regimen and duration of course of one of the susceptible antibiotics will be chosen and prescribed by Obstetrician to the participant, based on antibiotic susceptibility reported by the rapid test results.

**5. STUDY INTERVENTIONS**

**5.1 Interventions, Administration, and Duration**

The urine sample of the pregnant women enrolled in intervention arm will undergo rapid test screening for asymptomatic bacteriuria.

10 ml urine will be micro-filtered, and bacteria will be harvested in 3 ml of proprietary BITGEN medium which contains growth promotors for uropathogens, media cocktails and chromogens sensitive to bacterial growth. Four drops of this suspension will be added into a pre-functionalized identification strip and each well of two pre-loaded strips with antibiotics. The strips will then be incubated at 37°C for 4 hours for both identification and antibiotic sensitivity in a single go. The enzymatic hydrolysis of specific cocktails by uropathogens in the media optimized for promotion of their growth, leads to metabolizing of the chromogens and a change in color of the BITGEN media. Optical sensors will be used for measurement of all obtained color combinations. The direct quantitative identification of the common UTI-causing bacteria found in human urine, namely Escherichia coli, Klebsiella, Pseudomonas, Enterococcus, Proteus, and Staphylococcus sp. will be based on specific chromogenic endpoints produced. The first well of each antibiotic strip will show bacterial growth and the remaining 7 wells of each 8-welled strip will show varied levels of bacterial growth in presence of antibiotic, thus indicating the susceptibility of the bacteria to the antibiotic, reflected by a change in color of the BITGEN media. The two antibiotic strips will test for total 14 antibiotics Amoxicillin, Gentamicin, Amikacin, Cefepime, Ofloxacin, Ciprofloxacin, Ceftriaxone, Piperacillin-Tazobactum, Cefotaxime, Cefuroxime, Tobramycin, Levofloxacin, Cefazolin, and Imipenem. The concentration and composition of the antibiotics will be as per Clinical and Laboratory Standards Institute (CLSI) guidelines.

After incubation, the identification strip and two antibiotic strips will be mounted on the reader machine which will measure chromogenic and nephelometric endpoints produced due to change in color produced by the strips, using an array of 64 photodiodes in an electronic optical sensor prefabricated on the reader machine. The sensor output will be analyzed using a proprietary indigenous lab-developed statistical algorithm-based software, pre-installed on the reader. The results will include bacterial load, identification of the pathogen and its sensitivity to the panel of antibiotics. The software will also prompt for the need of additional incubation in case of insufficient growth due to low bacterial load.

The results will be shared with the obstetricians to prescribe any of the susceptible antibiotics.

**5.2 Handling of Study Interventions**

The reader machine will be installed in the microbiology laboratory of Gandhi Hospital for the entire period of the study. The strips and logistics required for the preparation of the sample including BITGEN media will be provided by BITS Pilani, Hyderabad Campus. The test will be conducted by a lab technician from BITS, who will also carry the required logistics with him/her every working day. The unused study products will be carried back to BITS Pilani.

The participants will not be aware of their allocation status unless they receive antibiotic for asymptomatic bacteriuria. The outcome assessors will be blinded for the allocation status of the participants.

**5.3 Concomitant Interventions**

No concomitant intervention is specified for this trial, although all the participants will be advised to maintain good personal hygiene and good diet by the obstetrician during antenatal checkup. For those participants put on antibiotics, any side effect arising out of the medication may warrant additional medication or life-saving measures depending upon the adverse event.

**5.4 Adherence Assessment**

A telephonic follow-up will be done with those participants who will be prescribed antibiotics, so as to ensure and assess adherence till they take all the pills as per the prescribed regimen. The adherence will be assessed based on the information shared by the participants about the remaining pills.

**6. STUDY PROCEDURES**

**6.1 Schedule of Evaluations**

| **Assessment** | **Screening, baseline enrollment, randomization: Visit-1** | **Telephonic Follow up 1 (Day 1)** | **Telephonic Follow up 2 (Day 3)** | **Telephonic Follow up 3 (Day 7)** | **Physical Follow up Visit (EDD around 9 months of pregnancy)** |
| --- | --- | --- | --- | --- | --- |
| Informed Consent Form | **X** |  |  |  |  |
| Demographics | **X** |  |  |  |  |
| Medical History | **X** |  |  |  |  |
| General Physical Examination | **X** |  |  |  |  |
| Current Medications | **X** |  |  |  |  |
| Blood Chemistries (As per antenatal checkup) | **X** |  |  |  |  |
| Hematology (As per antenatal checkup) | **X** |  |  |  |  |
| Vital Signs (As per antenatal checkup) | **X** |  |  |  |  |
| Inclusion/Exclusion Criteria | **X** |  |  |  |  |
| Enrollment/Randomization | **X** |  |  |  |  |
| Rapid Test | **X** |  |  |  |  |
| Treatment Administration | **X** |  |  |  |  |
| Telephonic follow-up |  | **X** | **X** | **X** |  |
| Adverse Events |  | **X** | **X** | **X** |  |
| Outcome Assessment |  |  |  |  | **X** |

**6.2 Description of Evaluations**

**6.2.1 Screening Evaluation**

**Consenting Procedure**

GCP guidelines will be followed for consenting procedure. A single informed consent form will describe both the screening and study procedures. The research staff at antenatal clinic will include an MD doctor and a lab technician. The doctor will conduct the consent process by explaining in English or the vernacular language (Telugu) the study procedure, purpose of the study, and choice of the participants throughout the study. The written informed consent form will be printed in the vernacular language as well as English. The participant will read the form and ask queries, which will be addressed by the doctor. The signed consent document will be maintained by the Principal Investigator.

**Screening**

The screening will be performed at the time of study entry. The history will be taken, and the participant will be assessed physically for any sign or symptom of UTI. The screening evaluation will include:

A. History Taking

a. History of recent antibiotic usage

b. History of symptoms of UTI

B. Physical Examination

a. Signs of hypovolemia

b. Temperature and blood pressure

C. Prior result of urine pregnancy test or ultrasound (for confirmation of pregnancy)

Only if the inclusion/exclusion criteria are met, will a participant be enquired about antenatal history of gravida and para, and her urine sample will be collected.

**6.2.2 Enrollment, Baseline, and/or Randomization**

**Enrollment**

After screening, the enrolled participants will be enquired about more history and the urine sample will be collected. The randomization sequence will be concealed from the enrolling doctor. The randomization will be done in microbiology laboratory by the Principal Investigator. The date of enrollment will be the date of first antenatal visit, when in the same visit, her urine sample is collected, history is taken, and she is randomized to one of the arms.

**Baseline Assessments**

1. History taking

2. Physical examination

3. Rapid test on urine sample in case of intervention arm

**Randomization**

Randomization will be done on the same day of screening, baseline, and urine sample collection. Randomization will be done as soon as the sample reaches microbiology laboratory. The allocation sequence like the one generated by MS excel random number generation feature will be concealed from the research staff who will enroll the participants.

**6.2.3 Blinding**

Blinding will be done for outcome assessors, who will be provided only identification details of the enrolled participants. Under no circumstance, will the blinding be broken. The outcome assessors will at least be graduates with some experience in field epidemiology.

**6.2.4 Followup Visits**

Although study will not require any additional follow up visit to the hospital, but the participants will be encouraged to come for regular antenatal checkups as scheduled by the obstetricians. The routine tests and assessment of antenatal checkups will be carried out as per the guidelines of the institute. In case of any adverse event or urinary tract infection, the participants will be shared contact number of the Principal Investigator and research staff, who will facilitate the care provision at the hospital.

Since the participants need not deliver in Gandhi Hospital itself, or may not necessarily deliver around the Expected Date of Delivery, a follow up visit will be conducted by outcome assessors to the hospital where they will be admit or at home if the delivery is already over. The coordination will be done around EDD on telephone.

The follow up visit should be completed by the outcome assessors around the delivery or as soon as possible. The outcome assessors will collect data on week of gestation at the time of delivery, birth weight, any maternal or perinatal co-morbidity encountered, and will collect the snapshot of the discharge card or treatment record as per the availability with the participant.

**6.2.5 Completion/Final Evaluation**

Any adverse event will be reported telephonically by the participant during telephonic follow-up. In case of symptomatic UTI, rapid test will be conducted for the participant again and antibiotic regimen will again be prescribed. No other evaluation is needed for repeat visit of the participant.

**7. SAFETY ASSESSMENTS**

In case any adverse event due to antibiotic is experienced, the antibiotic will be discontinued. In case UTI is experienced by participants of intervention as well as control arm, the participant will undergo repeat testing and will be given antibiotics, so as to progress the infection to pyelonephritis.

**7.1 Specification of Safety Parameters**

For symptomatic UTI experienced later in the course of pregnancy, temperature more than 99 F will be considered as fever. The result of rapid test will be regarded as final, although the obstetrician will be free to order a urine culture to confirm the report.

**7.2 Methods and Timing for Assessing, Recording, and Analyzing Safety Parameters**

At any time during the pregnancy after the enrollment in the study, if a participant experiences any side effect of antibiotics or signs and symptoms of UTI, the participant will call the Principal Investigator or research staff. The risk of encountering UTI is higher in second and third trimester [35].

**7.3 Adverse Events and Serious Adverse Events**

Although the study involves urine collection and prescription of commonly prescribed antibiotics which have been safely used since last many years, in case any participant experiences any adverse event due to consumption of antibiotics, the participant will be promptly transported to the hospital and managed accordingly. The adverse events and serious adverse events will be reported to the regulatory authorities and ethics committee, as well as to the sponsor immediately. The detailed reports will be submitted as per GCP guidelines.

In case the participant does not respond to an antibiotic labelled as susceptible by the rapid test, a confirmatory urine culture will be conducted on day three after the prescription.

**7.4** **Reporting Procedures**

Principal Investigator will be responsible to report any adverse event immediately to the sponsor and ethics committee. In case an adverse event occurs due to an antibiotic, the same will not be prescribed again to the participant. Since the antibiotics used by the obstetricians will be routinely used in clinical practice, the remaining participants will still be prescribed the antibiotics as per the report of the rapid test.

**7.5 Followup for Adverse Events**

The participant experiencing adverse event will be followed up throughout the remaining pregnancy once a week for any residual morbidity.

**7.6 Safety Monitoring**

The sponsor will conduct periodic monitoring visits to the trial site and BITS Pilani laboratory. Regular update of the trial progress will be shared with the funder in review meetings (frequency will be decided by the funder).

**8. INTERVENTION DISCONTINUATION**

The criteria for discontinuing the intervention for a participant:

1. If the participant experiences any adverse event due to an antibiotic prescribed, the participant can discontinue the regimen prescribed and seek medical help.

2. If the participant decides to no more be a part of the study and refuses to provide the information related to the outcome of pregnancy

The criteria for discontinuation of the intervention of rapid test:

1. If more than 10% participants report that the antibiotics prescribed based on the results of the rapid test could not arrest the disease process or turn symptomatic (although the rapid test has been validated in multicentric studies previously)

Even if the participants discontinue antibiotic regimen, they will be followed up for the outcomes of the pregnancy. Participants need to report any signs and symptoms of UTI if they discontinue the antibiotic regimen.

**9. STATISTICAL CONSIDERATIONS**

**9.1 General Design Issues**

The process of development of prematurity and low birth weight due to asymptomatic bacteriuria is largely insidious and multifactorial. Training status of lab technicians, resource availability, adherence to guidelines, myths among the providers regarding asymptomatic nature of the disease and lack of habit of conducting point-of-care tests in routine antenatal check-ups introduce variability at facility level. Hence, to study the effect of early diagnosis of asymptomatic bacteriuria and subsequent targeted therapy in real-world situation, pragmatic randomized controlled trial design was chosen. The primary hypothesis is that early diagnosis and treatment of asymptomatic bacteriuria will decrease preterm and low birth weight. The primary outcome measure is incidence of preterm and/or low birth weight in usual care of a setting and in an intervention arm with early diagnosis using rapid test and subsequent informed treatment with antibiotics. Hence parallel study design was chosen to prove the superiority of the intervention. Secondary hypothesis was that some co-morbidities can be confounders for preterm and low birth weight which are otherwise multifactorial. Hence, secondary outcome measure was the incidence of maternal and perinatal co-morbidities.

**9.2 Sample Size and Randomization**

Sample size was calculated based on type 1 error of 5%, power of 80%, global rate of preterm birth as 10.6% and that of low-birth-weight as 14.6%, combined rate of preterm or low-birth-weight (primary outcome measure) extrapolated as 25%. Based on the contribution of asymptomatic bacteriuria to total preterm birth and low-birth-weight, which varies from 6.9% to 20.5%24, the expected incidence of preterm birth or low-birth-weight in intervention arm for sample size calculation, was safely assumed to be 15% lower than the above-extrapolated global incidence of 25% in the control arm, as a minimally important difference for success of the experiment. Loss to follow up was accounted for 10% participants (5% in each arm). The sample size calculation estimated 120 participants to be enrolled in each arm, with a total 240 participants to be enrolled in the study.

Intention-to-treat analysis will be done in the original groups in which the participants are allocated. Incidence of other maternal and perinatal events will be described as frequencies. In case there is loss to follow up, complete case analysis will be done. In case there is any protocol deviation, per protocol analysis will be done.

**Treatment Assignment Procedures**

Randomization allocation sequence will be concealed from the participant enrollment team and will be maintained in the soft copy by the Principal Investigator, who will implement the randomization in a separate room in microbiology laboratory, and will discard the urine samples of the control arm.

No previous records will be shared with the blinded outcome assessors, who will be provided blank printed formats with only the identification details of the participants. No one will be authorized to break the blinding.

**9.3 Definition of Populations**

Intention to treat analysis will be considered when all the participants complete the study protocol and there is no loss to follow up. Per protocol population will be considered when any of the participant will not be able to complete the antibiotic regimen.

**9.4 Interim Analyses and Stopping Rules**

No interim analysis is proposed for the trial. The stopping rule will be when more than 10% participants report that the antibiotics prescribed based on the results of the rapid test could not arrest the disease process or turn symptomatic.

**9.5 Outcomes**

Outcomes will be analyzed by the Principal Investigator. The documentation of the outcome will be validated by the Principal Investigator based on the supporting medical records collected.

**9.5.1 Primary Outcome**

Primary outcome for the study is incidence of preterm birth and low birth weight. It will be measured by the outcome assessors at the time of follow up visit after delivery.

**9.5.2 Secondary Outcomes**

Secondary outcome is incidence of other maternal and perinatal morbidities and it will be measured during the same visit as the primary outcome.

**9.6 Data Analyses**

Pearson’s chi square will be used for analyzing data, after tabulation of categorical dichotomous variables in a 2x2 table, drawn between the outcome of preterm birth or low birth weight, and arm in which the incidence occurred.

**10. DATA COLLECTION AND QUALITY ASSURANCE**

**10.1 Data Collection Forms**

The research staff enrolling the participants in antenatal clinic will include an MD doctor and a lab technician. The identification details, inclusion/exclusion criteria, clinical history and examination details of signs and symptoms, and antenatal history will be recorded in a prespecified format by the doctor. The result of the rapid test will be entered by the Principal Investigator in the same format in the designated space.

The outcome assessors will be provided a separate format to record the outcomes. They will be provided only the identification details.

**10.2 Data Management**

Data management will be done by the Principal Investigator. The data collection forms are attached in the Appendix 3 and 4.

**10.3 Quality Assurance**

**10.3.1 Training**

The labor room staff at Gandhi Hospital will be trained for obstetric examination, weight measurement and estimation of gestational age once before the study.

The outcome assessors will be trained for interviewing the client and using the data collection format.

**10.3.2 Quality Control Committee**

NA

**10.3.3 Metrics**

NA

**10.3.4 Protocol Deviations**

Protocol deviation can occur in case any participant is not evaluated properly, or urine sample is not collected as per the guideline. All such incidences will be reported on the data collection format and will be reviewed by the Principal Investigator.

**10.3.5 Monitoring**

Once a week monitoring visit will be made by the Principal Investigator to antenatal clinic. The records and consent forms will be reviewed daily by the Principal Investigator. A sub-sample of 5% of delivered women will be revisited by the Principal Investigator at their home for outcome assessment.

**11. PARTICIPANT RIGHTS AND CONFIDENTIALITY**

**11.1 Institutional Review Board (IRB) Review**

This protocol and the informed consent document (Appendix 1) and any subsequent modifications will be reviewed and approved by the IRB or ethics committee responsible for oversight of the study. An IRB approval will be obtained by submitting proposal, formats, and this protocol.

**11.2 Informed Consent Forms**

A signed consent form will be obtained from each participant. In case a participant is unable to sign, thumb impression will be taken on the informed consent form after undergoing the consent procedure. In case a participant cannot consent herself, a legal guardian will be involved in consent procedure and the same will be documented.

**11.3 Participant Confidentiality**

The data of the participant will be anonymized by safe harbour procedure. All records will be kept in a secured cabinet and all computer entries will be password-protected. The participants will be assigned a serial number for identification.

**11.4 Study Discontinuation**

The study may be discontinued at any time by the IRB, the regulatory bodies, the sponsor, or other government agencies as part of their duties to ensure that research participants are protected.

**12. COMMITTEES**

NA

**13. PUBLICATION OF RESEARCH FINDINGS**

Any presentation, abstract, or manuscript will be made available for review by the sponsor prior to submission.

**14. REFERENCES**

1. Lindsay E. Nicolle, Suzanne Bradley, Richard Colgan, James C. Rice, Anthony Schaeffer, and Thomas M. Hooton “Infectious Diseases Society of America Guidelines for the Diagnosis and Treatment of Asymptomatic Bacteriuria in Adults” Clinical Infectious Diseases. 2005, 40:643–654.

2. Uncu Y., Uncu G., Esmer A., Bilgel N. “Should asymptomatic bacteriuria be screened in pregnancy?” Clinical and Experimental Obstetrics & Gynecology. 2002, 29(4):281-285.

3. Vaishali Jain, Vinita Das, Anjoo Agarwal & Amita Pandey “Asymptomatic bacteriuria & obstetric outcome following treatment in early versus late pregnancy in north Indian women” Indian Journal of Medical Research. April 2013, 137:753-758.

4. Gilstrap L. C. 3rd, Ramin S. M. “Urinary tract infections during pregnancy” Obstetrics & Gynecology Clinics of North America. 2001, 28(3):581.

5. Richard L. Naeye “Causes of the Excessive Rates of Perinatal Mortality and Prematurity in Pregnancies Complicated by Maternal Urinary-Tract Infections” New England Journal of Medicine. 1979, 300:819-823.

6. Okrągła E., Szychowska K., Wolska L. “Mechanisms of urinary tract sterility maintenance” Postępy Higieny i Medycyny Doświadczalnej. 2014 Jun 2, 68:684-694. [Article in Polish]

7. Humera Qudsia Fatima Ansari, Aruna Rajkumari “Prevalence of asymptomatic bacteriuria and associated risk factors among antenatal women attending a tertiary care hospital” Journal of Medical and Allied Sciences. 2011, 1(2):74- 78.

8. John E. Delzell, Michael L. Lefevre “Urinary Tract Infections during Pregnancy” American Family Physician. 2000 Feb 1, 61(3):713-720.

9. Nicolle LE. “Asymptomatic bacteriuria: when to screen and when to treat” Journal of Infectious Disease Clinics of North America. 2003, 17:367–394.

10. Lee, M., Bozzo, P., Einarson, A. & Koren, G. “Urinary tract infections in pregnancy” Canadian Family Physician. 2008, 54:853-854.

11. Andabati G., Byamugisha J. “Microbial aetiology and sensitivity of asymptomatic bacteriuria among antenatal mothers in Mulago Hospital, Uganda” African Health Sciences Journal. 2010, 10(4):349–352.

12. Akinloye O., Ogbolu D. O., Akinloye O. M., Terry Alli O. A. “Asymptomatic bacteriuria of pregnancy in Ibadan, Nigeria: a re-assessment” British Journal of Biomedical Science. 2006, 63:109-112.

13. Fatima N., Ishrat S. “Frequency and risk factors of asymptomatic bacteriuria during pregnancy” Journal of the College of Physicians and Surgeons Pakistan. 2006, 16: 273-275.

14. Smaill F. “Antibiotics for asymptomatic bacteriuria in pregnancy” Cochrane Database Systematic Review. 2001, 2:CD000490.

Available from:

http://apps.who.int/rhl/reviews/langs/CD000490.pdf

15. Gratacos E., Torres P-J, Vila J., Alonso P. L., Cararach V. “Screening and treatment of asymptomatic bacteriuria in pregnancy prevent-pyelonephritis” Journal of Infectious Diseases. 1994, 169:1390–1392.

16. Mittendorf R., Williams M. A., Kass E. H. “Prevention of preterm delivery and low birth weight associated with asymptomatic bacteriuria” Clinical Infectious Diseases. 1992, 14:927–932.

17. Romero R., Oyarzun E., Mazor M., Sirtori M., Hobbins J. C., Bracken M. “Meta-analysis of the relationship between asymptomatic bacteriuria and preterm delivery/low birth weight” Obstetrics & Gynecology Journal. 1989; 73:576–582.

18. U.S. Preventive Services Task Force “Screening for Asymptomatic Bacteriuria in Adults: U.S. Preventive Services Task Force Reaffirmation Recommendation Statement” Annals of Internal Medicine. 2008,149:43-47.

19. Joy D. Van Nostrand, Alan D. Junkins, Roberta K. Bartholdi “Poor Predictive Ability of Urinalysis and Microscopic Examination to Detect Urinary Tract Infection” American Journal of Clinical Pathology. 2000,113:709-713.

20. Mathai E., Thomas R. J., Chandy S., Mathai M., Bergstrom S. “Antimicrobials for the treatment of urinary tract infection in pregnancy: practices in southern India” Pharmacoepidemiology and Drug Safety. 2004 Sep, 13(9):645-652.

21. Aigere E. O., Okusanya B. O., Eigbefoh J. O., Okome G. B. “Enhanced urinalysis in the detection of asymptomatic bacteriuria in pregnancy” Nigerian Quarterly Journal of Hospital Medicine. 2013 Apr-Jun, 23(2):105-109.

22. Okusanya B. O., Aigere E. O., Eigbefoh J. O., Okome G. B., Gigi C. E. “Is a chlorhexidine reaction test better than dipsticks to detect asymptomatic bacteriuria in pregnancy?” Obstetrics & Gynecology Journal. 2014 Jan, 34(1):21-24.

23. Awonuga D. O., Fawole A. O., Dada-Adegbola H. O., Olola F. A., Awonuga O. M. “Asymptomatic bacteriuria in pregnancy: evaluation of reagent strips in comparison to microbiological culture” African Journal of Medicine and Medical Sciences. 2011 Dec, 40(4):377-383.

24. Ullah A., Barman A., Ahmed I., Salam A. J. “Asymptomatic bacteriuria in pregnant mothers: a valid and cost-effective screening test in Bangladesh” Obstetrics & Gynecology Journal. 2012 Jan, 32(1):37-41.

25. Lumbiganon P., Laopaiboon M., Thinkhamrop J. “Screening and treating asymptomatic bacteriuria in pregnancy” Current Opinion in Obstetrics and Gynecology. 2010 Apr, 22(2):95-99.

26. Leman P. “Validity of urinalysis and microscopy for detecting urinary tract infection in the emergency department” European Journal of Emergency Medicine. 2002 Jun, 9(2):1417.

27. Greeff A., Jeffery B., Pattinson R. C. “Uricult trio as a screening test for bacteriuria in pregnancy” South African Medical Journal. 2002 Apr, 92(4):306-309.

28. Shelton S. D., Boggess K. A., Kirvan K., Sedor F., Herbert W. N. “Urinary interleukin-8 with asymptomatic bacteriuria in pregnancy” Obstetrics & Gynecology Journal. 2001 Apr, 97(4):583-586.

29. Mathews J. E., George S., Mathews P., Mathai E., Brahmadathan K. N., Seshadri L. “The Griess test: an inexpensive screening test for asymptomatic bacteriuria in pregnancy” Australian and New Zealand Journal of Obstetrics and Gynaecology. 1998 Nov, 38(4):407-410.

30. de Wet A., Louw N. S. “Assessing the incidence of asymptomatic bacteriuria in pregnancy by means of the Dornfest method as a screening test” South African Medical Journal. 1982 Aug 21, 62(9):285. [Article in Afrikaans]

31. Archbald F. J., Verma U., Tejani N. A. “Screening for asymptomatic bacteriuria with Microstix” The Journal of Reproductive Medicine. 1984 Apr, 29(4):272-274.

32. Bilir F., Akdemir N., Ozden S., Cevrioglu A. S., Bilir C. “Increased serum procalcitonin levels in pregnant patients with asymptomatic bacteriuria” Annals of Clinical Microbiology and Antimicrobials. 2013 Sep 5, 12:25.

33. Jayalakshmi J., Jayaram V. S. “Evaluation of various screening tests to detect asymptomatic bacteriuria in pregnant women” Indian Journal of Pathology & Microbiology. 2008, 51(3):379-381.

34. Gayathree L., Shetty S., Deshpande S. R., Venkatesha D. T. “Screening For Asymptomatic Bacteriuria in Pregnancy: An Evaluation of Various Screening Tests in Hassan District Hospital, India” Journal of Clinical and Diagnostic Research. 2010 Aug, 4(4):2702-2706.

35. Dawkins JC, Fletcher HM, Rattray CA, Reid M, Gordon-Strachan G. Acute Pyelonephritis in Pregnancy: A Retrospective Descriptive Hospital Based-Study. ISRN Obstet Gynecol [Internet]. 2012 [cited 2020 Nov 8]; 2012:1–6. Available from: /pmc/articles/PMC3505646/

**15. SUPPLEMENTS/APPENDICES**

**Appendix 1 Informed Consent Form (English)**

| **WRITTEN INFORMED CONSENT FORM FOR MOTHERS** |
| --- |

**Study Title: Evaluation of rapid strip test for screening bacteriuria among pregnant women and its effect on incidence of low birth weight and prematurity in new-borns**

**Principal Investigator:** Dr K Nagamani, Dr T S Usha Sree, Prof. Suman Kapur and Dr. Manish Gehani, ID No-2013PHXF500H (BITS Pilani, Hyderabad Campus)

**What you should know about this study**

- You are being asked to participate in a research study.
- This consent form explains the research study and your part in the study.
- You are a volunteer. You can choose not to take part and if you join, you may quit at any time. There will be no penalty if you decide to quit the study.

**Purpose of research project**

| Rapid strip test is a novel test to provide urine culture report for urinary tract infections in approximately four hours instead of conventional test which gives result in 18 to 48 hours. This easy-to-use bedside rapid test helps doctors prescribe appropriate antibiotic and helps prevent antibiotic resistance. The informed prescription will help prevent the discomfort to the mother and the complications during pregnancy due to urinary tract infections. The study is being supported by National Programme on Micro and Smart Systems (NPMASS), the Department of Microbiology, Gandhi Medical College, Hyderabad and BITS Pilani, Hyderabad Campus.  The purpose of this initiative is to improve antibiotics prescription in urinary tract infections and to cut short the waiting time for the result of urine culture test. The study also aims to diagnose asymptomatic cases which may suffer from discomfort and complications during the pregnancy if otherwise undiagnosed. |
| --- |

**Why you are being asked to participate**

| You have been selected to take part in this study as you are attending antenatal checkup or outpatient treatment in a facility (hospital) where this initiative is piloted. |
| --- |

**PROCEDURES**

| If you join this study, your urine sample will be collected and tested with a rapid strip test and a urine culture test and your data will be shared with the program team for further analysis. |
| --- |

**RISKS/DISCOMFORTS**

| There is no risk or discomfort involved in this process. We will not show the information to anyone else. |
| --- |

**BENEFITS**

| *Benefits to you*   - You may receive no direct benefit from the study. - In the future, pregnant women will get timely reporting for urinary tract infections and there may be a reduction in adverse outcomes of the same during pregnancy. |
| --- |

**Payment**

| You will not receive any payment or compensation for participating in the study. |
| --- |

**Protecting data confidentiality**

| We will write your name in the study forms alone. The data will be kept confidential. No one at this health facility/ site will get to see the information that we collect. |
| --- |

**What happens if you leave the study early?**

| You are free to participate or not. If you agree to participate, you can change your mind and ask the interviewer to end the interview at any time. If you decide not to participate, this will not affect your service today or in the future. |
| --- |

**Who do I call if I have questions or problems?**

1. Dr. Manish Gehani
2. Program Officer
3. Jhpiego (An affiliate to Johns Hopkins University)
4. 2nd Floor
5. Indian Institute of Health and Family Welfare,
6. Opp –Nalanda Junior College
7. Near SR Nagar Cross Roads
8. Vengalrao Nagar
9. Hyderabad-500038, Telangana, India
10. Tel: + 917568874444

**Permission to conduct this study has been granted from:**

1. Gandhi Medical College, Hyderabad

**PERMISSION TO PROCEED**

I attest that I understand the content of this consent form and voluntarily agree to participate in this initiative.

**__________________________ ___________________**

Signature/thumb impression of the mother: Date:

**Thank you for your time and assistance**

**Appendix 2 Informed Consent Form (Telugu)**

| **తల్లుల కొరకు రాతపూర్వక సమ్మతి పత్రం** |
| --- |

**అధ్యయన శీర్షిక: గర్భవతులైన మహిళ్లల మూత్రంలో సూక్ష్మజీవులు ఉండటం కొరకు స్క్రీనింగ్ కొరకు ర్యాపిడ్ స్ట్రిప్ టెస్ట్ మదింపు మరియు తక్కువ జనన బరువు మరియు నవజాత శిశువుల్లో బరువు తక్కువగా ఉండే ఘటనలపై ప్రభావం.**

**ప్రధాన పరిశోధకుడు:** డాక్టర్. కె. నాగమణి, డాక్టర్. టిఎస్ ఉషశ్రీ, ప్రొఫెసర్ సుమన్ కపూర్ మరియు డాక్టర్ మనీష్ గెహానీ, ఐడి నెంబరు- 2013PHXF500H (BITS పిలానీ, హైద్రాబాద్ క్యాంపస్)

**ఈ అధ్యయనం గురించి మీరు ఏమి తెలుసుకోవాలి?**

- ఒక పరిశోధన అధ్యయనంలో పాల్గొనేందుకు మీరు కోరబడ్డారు.
- ఈ సమ్మతి పత్రం పరిశోధన అధ్యయనం మరియు దీనిలో పాల్గొనడం గురించి వివరిస్తుంది.
- మీరు ఒక స్వచ్చంధకుడు. మీరు పాల్గొనరాదని ఎంచుకోవచ్చు లేదా ఒకవేళ చేరినట్లయితే, ఏ సమయంలోనైనా మీరు నిష్క్రమించవచ్చు. అధ్యయనం నుంచి మీరు నిష్క్రమించాలని అనుకుంటే ఎలాంటి అపరాధ రుసుం ఉండదు.

**అధ్యయన ప్రాజెక్ట్ ఉద్దేశ్యం**

| 18 నుంచి 48గంటలు పట్టే సంప్రదాయ టెస్ట్‌కు బదులుగా సుమారు నాలుగు గంటల్లోనే మూత్ర నాళ సంక్రామ్యతల కొరకు యూరిన్ కల్చర్ రిపోర్ట్ అందించడానికి ర్యాపిడ్ స్ట్రిప్ టెస్ట్ అనేది ఒక వినూత్న పరీక్ష. తేలికగా బెడ్ వద్ద ఉపయోగించే ర్యాపిడ్ టెస్ట్, తగిన యాంటీబయోటిక్‌ని సిఫారసు చేయడానికి మరియు యాంటీబయోటిక్ నిరోధకత్వం తగ్గించడానికి దోహదపడుతుంది. మూత్రనాళ సంక్రామ్యతల వల్ల గర్భధారణ సమయంలో తల్లులకు కలిగే అసౌకర్యం మరియు సంక్లిష్టతలను నిరోధించడానికి వివేచనతో కూడిన ప్రిస్కిప్షన్ సహాయపడుతుంది. అధ్యయనానికి నేషనల్ ప్రోగ్రామ్ ఆన్ మైక్రో అండ్ స్మార్ట్ సిస్టమ్స్ (NPMASS), డిపార్ట్‌మెంట్ ఆఫ్ మైక్రోబయాలజీ, గాంధీ మెడికల్ కాలేజీ, హైద్రాబాద్ మరియు BITS పిలానీ, హైద్రాబాద్ క్యాంపస్‌లు మద్దతు అందిస్తున్నాయి.  మూత్రనాళ సంక్రామ్యతల్లో యాంటీబయోటిక్స్ ప్రిస్కిప్షన్‌ని మెరుగుపరచడానికి మరియు యూరిన్ కల్చర్ టెస్ట్ ఫలితాల కొరకు వేచి ఉండే సమయాన్ని తగ్గించడమే ఈ ప్రోత్సాహం ఉద్దేశ్యం. ఒకవేళ మరోవిధంగా నిర్ధారించబడకుండా ఉన్నట్లయితే, గర్భధారణ సమయంలో ఎదుర్కొనే అసౌకర్యం లేదా సంక్లిష్టతలతో బాధపడే వ్యాధిలక్షణ రహిత కేసులను నిర్ధారించడం కూడా అధ్యయనం లక్ష్యమే. |
| --- |

**పాల్గొనాలని మీరు ఎందుకు అడగబడ్డారు**

| మీరు గర్భధారణ పరీక్షలకు హాజరువుతున్నారు లేదా ఈ ప్రోత్సహాక కార్యక్రమం పైలట్ కార్యక్రమం నిర్వహిస్తున్న ఫెసిలిటీ(ఆసుపత్రి)లో అవుట్‌పేషెంట్‌గా చికిత్స పొందుతుండటంతో ఈ అధ్యయనంలో పాల్గొనేందుకు మీరు ఎంచుకోబడ్డారు. |
| --- |

**ప్రక్రియలు**

| ఒకవేళ మీరు ఈ అధ్యయనంలో పాల్గొన్నట్లయితే, మీ మూత్ర నమూనా సేకరించబడుతుంది మరియు ర్యాపిడ్ స్ట్రిప్ టెస్ట్ ద్వారా పరీక్షించబడుతుంది మరియు యూరిన్ కల్చర్ టెస్ట్ కూడా నిర్వహించబడుతుంది, తదుపరి విశ్లేషణ కొరకు మీ డేటా ప్రోగ్రామ్ టీమ్‌తో పంచుకోబడుతుంది. |
| --- |

**ప్రమాదాలు/అసౌకర్యాలు**

| ఈ ప్రక్రియలో ఎలాంటి ప్రమాదం లేదా అసౌకర్యం ఉండదు. మేం సమాచారాన్ని ఎవరికి చూపించం. |
| --- |

**లాభాలు**

| *మీకు లాభాలు*   - అధ్యయనం నుంచి మీరు ప్రత్యక్ష ప్రయోజనాలు పొందకపోవచ్చు. - భవిష్యత్తులో, గర్భవతులైన మహిళలు మూత్రనాళ సంక్రామ్యతలను సకాలంలో నివేదించగలుగుతారు మరియు గర్భధారణ సమయంలో ప్రతికూల ఫలితాలు తగ్గవచ్చు. |
| --- |

**చెల్లింపు**

| ఈ అధ్యయనంలో పాల్గొన్నందుకు మీరు ఎలాంటి ప్రతిఫలం లేదా పారితోషికాన్ని పొందరు. |
| --- |

**డేటా గోప్యతను సంరక్షించడం**

| అధ్యయన ఫారాల్లోనే మీ పేరు రాయబడుతుంది. డేటా గోప్యంగా ఉంచబడుతుంది. మేం సేకరించిన సమాచారాన్ని మీ వైద్య సదుపాయం/ సైట్‌లోని ఎవరూ చూడరు. |
| --- |

**అధ్యయనాన్ని మీరు ముందస్తుగా విడిచిపెట్టినట్లయితే ఏమి జరుగుతుంది?**

| పాల్గొనాలా లేదా అనే స్వేచ్ఛ మీకుంటుంది. పాల్గొనాలని మీరు అంగీకరించినట్లయితే, మీరు మీ మనస్సుమార్చుకోవచ్చు మరియు ఏదైనా సమయంలో ఇంటర్వ్యూ ముగించమని ఇంటర్వూ చేసేవారిని అడగవచ్చు. ఒకవేళ మీరు పాల్గొనరాదని నిర్ణయించుకున్నట్లయితే, ఇవాళ లేదా భవిష్యత్తులో మీ సేవలపై ఎలాంటి ప్రభావం పడదు. |
| --- |

**నాకు ప్రశ్నలు లేదా సమస్యలున్నట్లయితే నేను ఎవరికి కాల్ చేయాలి?**

1. డాక్టర్. మహేష్ గెహాని
2. ప్రోగ్రామ్ ఆఫీసర్
3. Jhpiego (జాన్స్ హాప్‌కిన్స్ యూనివర్సిటీ అఫిలియేషన్)
4. 2వ అంతస్థు
5. ఇండియన్ ఇనిస్టిట్యూట్ ఆఫ్ హెల్త్ అండ్ ఫ్యామిలీ వెల్‌ఫేర్,
6. నలందా జూనియర్ కాలేజీ ఎదురుగా,
7. ఎస్‌ఆర్ నగర్ క్రాస్ రోడ్స్ దగ్గర
8. వెంగళరావు నగర్
9. హైద్రాబాద్ -500038, తెలంగాణ, ఇండియా
10. టెలి: + 917568874444

**ఈ అధ్యయనం నిర్వహించడానికి అనుమతి మంజూరు చేసినవారు:**

1. గాంధీ మెడికల్ కాలేజీ, హైద్రాబాద్

**కొనసాగడానికి అనుమతి**

ఈ సమ్మతి పత్రంలో వివరాలను నేను అర్థం చేసుకున్నట్లుగా నేను సంతకం చేస్తున్నాను మరియు ఈ ప్రోత్సాహక కార్యక్రమంలో పాల్గొనేందుకు నేను స్వచ్చందంగా అంగీకరిస్తున్నాను.

**__________________________**  **___________________**

తల్లి సంతకం/బొటనవేలు ముద్ర తేదీ:

**మీ సమయానికి మరియు సాయానికి ధన్యవాదాలు**

**Appendix 3 Data Collection Format for Study Enrollment and Record of Laboratory Reports**

| **RECORD OF ENROLLED MOTHERS** | | | | |
| --- | --- | --- | --- | --- |
| **DATE OF EVALUATION-** | |  | **OPD REGISTRATION NUMBER-** | |
| **ANC NUMBER-** | |  | **LAB NUMBER-** | |
|  | |  |  | |
| **A. Identification Data** | |  | **D. Enrolment details** | |
| **Name-** | |  | **Arm** **of** **the** **study** **for** **which** **woman** **is** **enrolled** | ❑ **Asymptomatic** |
| **Husband’s** **name-** | |  |  | ❑ **Symptomatic** |
| **Address-** | |  | **ENROLMENT NUMBER-** | |
|  |  |  |  | |
| **Mobile/phone** **number-** | |  | **E. Demographic and socioeconomic profile** | |
| **Alternate** **number** **if** **available-** | |  | **Age-** | |
|  | |  | **Religion-** | |
| **B. Exclusion Criteria** | |  | **Education-** | |
| **Has** **pregnant** **woman** **received** **any** **antibiotics** **in** **last** **two** **weeks?** | ❑ **Yes** |  |  | |
|  | ❑ **No** |  | **F. Antenatal data** | |
|  | |  | **No** **of** **previous** **ANC** **visits-** | |
| **Is** **woman** **pregnant** **for** **more** **than** **38** **weeks?** | ❑ **Yes** |  | **Gestational** **age** **in** **weeks-** | |
|  | ❑ **No** |  | **Number** **of** **previous** **pregnancies-** | |
|  | |  | **Number** **of** **abortions-** | |
| **Was** **she** **tested** **for** **Rapid** **Strip** **test** **in** **any** **arm** **of** **study** **earlier?** | ❑ **Yes** |  | **Number** **of** **live** **children-** | |
|  | ❑ **No** |  |  | |
|  | |  | **Was** **she** **tested** **or** **treated** **for** **UTI** **earlier** **in** **this** **pregnancy?** | ❑ **Yes** |
|  | |  |  | ❑ **No** |
| ***Enrolled in the study?*** | ❑ **Yes** |  |  | |
|  | ❑ **No** |  | **G. Study Seal** | |
|  | |  |  | |
| **C. Symptoms- (Tick whichever are applicable-one or**  **more)** | |  |  |  |
| ***Cystitis*** | |  |  |  |
| **Going** **for** **passing** **urine** **frequently** | ❑ |  |  |  |
| **Feel** **like** **urgently** **passing** **urine** | ❑ |  |  |  |
| **Pain/burning** **sensation** **during** **passing** **urine** | ❑ |  |  |  |
| **Urge** **to** **pass** **urine** **at** **night** | ❑ |  |  |  |
| **Lower** **abdominal** **pain** | ❑ |  |  |  |
| **Low-back** **pain** | ❑ |  |  |  |
| **Bleeding** **in** **urine** | ❑ |  |  |  |
| ***Pyelonephritis*** | |  |  |  |
| **Pain** **on** **the** **side** **of** **the** **abdomen** | ❑ |  |  |  |
| **Nausea** | ❑ |  |  |  |
| **Vomiting** | ❑ |  | **H. Outcome of pregnancy** | |
| **Fever** **(>38ºC)** | ❑ |  | **Birth Weight-** | |
| **Costo-vertebral** **angle** **tenderness** | ❑ |  | **Gestational age at delivery-** | |
| **Chills** | ❑ |  | **Any complication/death-** |  |
| ***Asymptomatic*** | |  |  |  |
| **No** **symptoms** | ❑ |  |  |  |

**Appendix 4 Data Collection Format for Outcome Assessors**

Field Data Collection Checklist

| Date of visit |  |
| --- | --- |
| Enrolment number |  |
| ANC Number |  |
| IPD Number |  |
| Name |  |
| Alias/AKA |  |
| Age |  |
| Husband’s Name |  |
| Education |  |
| Did you sign any telugu form at Gandhi Hospital before pregnancy | □Yes □No  □I Don’t remember |
| Date of Delivery |  |
| Name and address of the hospital where she delivered |  |
| Gender of the baby |  |
| Weight of the baby at birth |  |
| Other outcomes/complications/ death during pregnancy/delivery/ postpartum(baby/mother) |  |
| Documents whose pic is collected | □Discharge card □Indoor ticket □Delivery Note □Baby note  □Other-Specify-______________ |
| Signature of Mother |  |
